# Enrichment of Phosphatidylethanolamine in Viral Replication Compartments via Co-opting the Endosomal Rab5 Small GTPase by a Positive-Strand RNA Virus

**DOI:** 10.1371/journal.pbio.2000128

**Published:** 2016-10-19

**Authors:** Kai Xu, Peter D. Nagy

**Affiliations:** Department of Plant Pathology, University of Kentucky, Lexington, Kentucky, United States of America; Institut Pasteur, France

## Abstract

Positive-strand RNA viruses build extensive membranous replication compartments to support replication and protect the virus from antiviral responses by the host. These viruses require host factors and various lipids to form viral replication complexes (VRCs). The VRCs built by Tomato bushy stunt virus (TBSV) are enriched with phosphatidylethanolamine (PE) through a previously unknown pathway. To unravel the mechanism of PE enrichment within the TBSV replication compartment, in this paper, the authors demonstrate that TBSV co-opts the guanosine triphosphate (GTP)-bound active form of the endosomal Rab5 small GTPase via direct interaction with the viral replication protein. Deletion of Rab5 orthologs in a yeast model host or expression of dominant negative mutants of plant Rab5 greatly decreases TBSV replication and prevents the redistribution of PE to the sites of viral replication. We also show that enrichment of PE in the viral replication compartment is assisted by actin filaments. Interestingly, the closely related Carnation Italian ringspot virus, which replicates on the boundary membrane of mitochondria, uses a similar strategy to the peroxisomal TBSV to hijack the Rab5-positive endosomes into the viral replication compartments. Altogether, usurping the GTP-Rab5–positive endosomes allows TBSV to build a PE-enriched viral replication compartment, which is needed to support peak-level replication. Thus, the Rab family of small GTPases includes critical host factors assisting VRC assembly and genesis of the viral replication compartment.

## Author Summary

Plants, animals, and humans are threatened by positive-stranded RNA viruses, which are one of the major groups of intracellular pathogens. To support robust virus replication, these viruses subvert intracellular membranes and co-opt host proteins into virus-induced replication compartments. Tomato bushy stunt virus (TBSV) is a model virus used in yeast to dissect the roles of lipids and proteins in virus replication. In this work, the authors show that one of the two TBSV replication proteins interacts with the guanosine triphosphate (GTP)-bound Rab5 small GTPase, which allows the virus to take advantage of phosphatidylethanolamine (PE)-rich endosomes to build viral replication compartments consisting of peroxisomes. Peak level of TBSV replication depends on the co-opted abundant PE-rich Rab5-positive membranes in plants, too.

## Introduction

All RNA viruses with positive-strand genomes replicate in close association with subcellular membranes in plant or animal cells. The virus-induced membranous structures representing the viral replication compartment help sequester viral proteins, viral RNAs, and co-opted host factors in confined areas for efficient viral replication complex (VRC) assembly and robust viral RNA replication, while also protecting the viral RNA from cellular defense mechanisms. Many viruses orchestrate membrane deformations, leading to generation of vesicle-like membrane invaginations with narrow openings towards the cytosol that harbor VRCs [1,2]. Current virology research is aimed at gaining deeper insights into the formation of viral replication compartments (frequently called replication organelles), which depends on interaction of viral replication proteins with subcellular membranes, lipids, and various co-opted host factors.

The fascinating new picture emerging with tombusviruses is the complex rearrangements of cellular membranes, alteration of metabolic processes, and recruitment of a surprisingly large number of host proteins for novel proviral functions. Tomato bushy stunt virus (TBSV) usurps cellular membrane remodeling proteins, including the endosomal sorting complex required for transport (ESCRT) machinery [3–5], sterols, and phospholipids, to induce an elaborate membranous replication compartment harboring numerous vesicle-like structures in peroxisomal boundary membranes that support robust tombusvirus replication in a protective microenvironment [6].

Genome-wide screens in connection with proteome-wide studies and lipidomics have revealed the possible roles of hundreds of host proteins in tombusvirus replication [7–10]. In addition to the recruitment of host proteins, tombusviruses also take advantage of various cellular lipids for VRC assembly and regulation of virus replication [11]. For example, phosphatidylethanolamine (PE) is essential for the formation of VRCs in an in vitro replicase assembly assay [12] and affects tombusvirus replication in vitro, in yeast and plant cells. PE and phosphatidylcholine (PC) are also important for the replication protein-driven recruitment of viral RNA into membranous VRCs and for the activation of the RdRp function of viral p92 replication protein [12,13].

Previous high throughput screens revealed that Rab5 (Ypt52, Ypt53, and Vps21 in yeast) and associated proteins, such as Gdi1 (Rab GTPase-binding protein) and Vps41 (a member of the HOPS complex), affected TBSV replication in yeast [10,14,15]. Rab5/Vps21 is a key regulator of early endosomal biogenesis, maturation, and homotypic and heterotypic fusions. The early endosomes obtain their cargoes, such as endocytic vesicles, from the Trans-Golgi network or from the plasma membrane [16]. Activation of Rab5 requires binding to the target membrane (endosomal vesicles and early endosome) via its prenyl anchor and interaction with guanine nucleotide exchange factor (GEF) [17], which promotes nucleotide exchange from guanosine diphosphate (GDP) to GTP. The activated GTP-loaded form of Rab5 then recruits effectors, which promote the formation of late endosomes (also called multivesicular bodies) via fusions and generation of intraluminar vesicles. The model plant *Arabidopsis thaliana* has three Rab5 orthologs. AtRab5A (RHA1) and AtRab5B (ARA7) show similar domain organization and C-terminal prenylation with the human Rab5C and the yeast Vps21, whereas AtRab5C (ARA6) is unusual, with N-terminal palmitoylation and N-myristoylation [18,19].

Among the many open questions regarding tombusvirus replication is the pathway/mechanism of efficient PE enrichment at the sites of replication. A previous study based on a genetic approach in yeast indicated that the PE-synthesizing enzymes could be separately deleted without detrimental effects on tombusvirus replication [12]. Therefore, these enzymes might play overlapping roles, or PE enrichment depends on intracellular redistribution of PE in cells.

In this paper, we have discovered an unexpected connection between the Rab5-positive endosomes and tombusvirus replication. Specifically, tombusviruses interact with Rab5 small GTPase that results in recruitment of endosomal lipids, most importantly PE, to peroxisomes or mitochondria for different tombusviruses. Altogether, this work demonstrates a new critical proviral function for Rab5 in generation of PE-enriched viral replication compartments.

## Results

### Tombusviruses Recruit the Endosomal Rab5 Small GTPase into the Viral Replication Compartment in Yeast Cells

**Fig 1:**
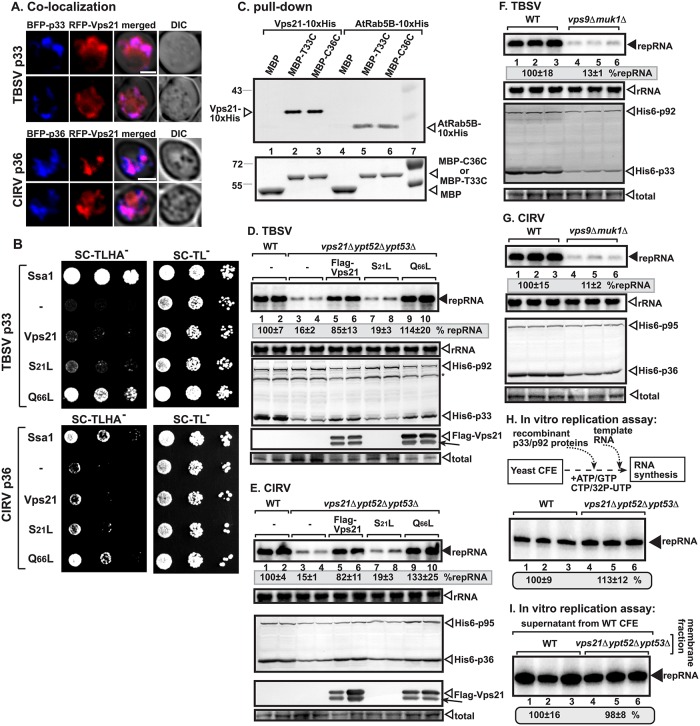
Interaction between p33 replication protein and yeast Rab5 ortholog Vps21p. (A) Confocal laser microscopy images show the partial co-localization of TBSV BFP-tagged p33 or the CIRV BFP-tagged p36 replication proteins with the RFP-tagged Vps21p protein in wt yeast cells. Differential interference contrast (DIC) images are shown on the right. Scale bars represent 2 μm. (B) The split ubiquitin assay was used to test binding between TBSV p33 or CIRV p36 and Vps21p in wt (NMY51) yeast. The bait p33/p36 was co-expressed with the shown prey proteins. The mutant Vps21Q_66_L is locked into the active GTP-bound stage, whereas the mutant Vps21S_21_L is locked into the inactive GDP-bound form. *SSA1* (HSP70 chaperone) and the empty prey vector (NubG) were used as positive and negative controls, respectively. The left panel shows p33:Vps21p interactions; the right panel demonstrates that comparable amounts of yeasts were used for these experiments. (C) Pull-down assay including the 10xHis-tagged yeast Vps21 and the *Arabidopsis* Rab5B proteins with the C-terminal (soluble) portion of the TBSV p33 (T33C) and CIRV p36 (C36C) replication proteins. Top panel: western blot analysis of the captured cellular proteins with the maltose-binding protein (MBP)-affinity purified p33C/p36C was performed with anti-His antibody. The negative control was MBP. Lane 7 shows molecular weight markers. Bottom panel: comassie blue-staining of protein gel with the purified MBP-p33C (the C-terminal soluble portion of TBSV p33), MBP-p36C (the C-terminal soluble portion), and MBP. (D) Decreased TBSV repRNA accumulation in *vps21Δypt52Δypt53Δ* yeast. To launch TBSV repRNA replication, we expressed His_6_-p33 and His_6_-p92 from the galactose-inducible *GAL1* promoter, and DI-72(+) repRNA from the galactose-inducible *GAL10* promoter in the parental (BY4741) and in *vps21Δypt52Δypt53Δ* yeast strains. FLAG-tagged Vps21 or its mutants were expressed from the copper-inducible *CUP1* promoter. The yeast cells were pre-cultured for 12 h at 29°C in 2% glucose SC minimal media, and then for 22 h at 23°C in 2% galactose SC minimal media supplemented with 50 μM CuSO_4_. Northern blot analysis was used to detect DI-72(+) repRNA accumulation. The accumulation level of DI-72(+) repRNA was normalized based on 18S rRNA levels (second panel from top). Bottom panels: western blot analysis of the accumulation level of His_6_-tagged p33, His_6_-p92, and FLAG-Vps21 proteins using anti-His and anti-FLAG antibodies, respectively. Note that FLAG-Vps21 forms a double band due to prenylation (a lipidation type of posttranslational modification) that is required for binding to the endosomal membrane. The faster migrating band represents the prenylated form of Vps21 (depicted by an arrow), whereas the unmodified form is depicted by an open arrowhead. The total protein samples were stained with coomassie blue. Each experiment was performed three times. (E) Decreased CIRV repRNA accumulation in *vps21Δypt52Δypt53Δ* yeast. See further details in panel D. (F) Decreased TBSV repRNA accumulation in *vps9Δmuk1Δ* yeast. See further details in panel D. (G) Decreased CIRV repRNA accumulation in *vps9Δmuk1Δ* yeast. See further details in panel D. (H) Comparable activities of the tombusvirus replicases assembled in cell-free extracts (CFEs) prepared from either wt or from *vps21Δypt52Δypt53Δ* yeast. Denaturing PAGE analysis of in vitro tombusvirus replicase activity in the CFEs. Note that this image shows the repRNAs made by a full cycle of replicase activity, producing both (-) and (+)-strands in vitro. The CFEs contained the same amount of total yeast proteins. Each experiment was performed three times. (I) Comparable activities of the tombusvirus replicases assembled in CFE membrane fractions prepared from either wt or from *vps21Δypt52Δypt53Δ* yeast. Note that the supernatant fraction was obtained from wt yeast CFE in each sample. See further details in panel H.

To gain insight into the putative role of Rab5 small GTPase in TBSV replication, we co-expressed red fluorescent protein (RFP)-tagged Vps21 (the major Rab5 protein in yeast) [20] with blue fluorescent protein (BFP)-tagged p33 replication protein in wild-type (wt) yeast cells, followed by confocal imaging. These experiments revealed partial co-localization of p33 and Vps21 (Fig 1A). Similar experiments with the closely related Carnation Italian ringspot virus (CIRV, which replicates on the outer mitochondrial membranes) p36 replication protein also showed partial co-localization with RFP-Vps21 in yeast cells (Fig 1A).

Because the above experiments indicated that Rab5 might be recruited to the viral replication compartment, we tested interaction between the replication proteins and the yeast Vps21p using membrane yeast two-hybrid (MYTH) assay [21]. Interestingly, strong interaction was detected between the p33 replication protein and a mutant Vps21Q_66_L that is locked into the active (GTP-bound) stage [22], while the wt Vps21p or a mutant Vps21S_21_L that is locked into the inactive (GDP-bound) form showed reduced interaction with p33 in the MYTH assay (Fig 1B). The experiments with the CIRV p36 replication protein showed comparable results, demonstrating strong interaction with the constantly active Vps21Q_66_L mutant (Fig 1B). Pull-down experiments with purified recombinant replication proteins also supported direct interaction between the C-terminal, cytosol-exposed region of the tombusvirus replication proteins, and Vps21p (Fig 1C, lanes 2–3). Altogether, these data support a model that the tombusvirus replication proteins directly interact with the active form of Rab5/Vps21p.

### Deletion of Rab5 Inhibits Viral Replication in Yeast

To test if Rab5 provides an important function during tombusvirus replication, we generated a yeast strain lacking all three Rab5 genes (*vps21Δ ypt52Δ ypt53Δ*) [23] and measured TBSV or CIRV repRNA levels by northern blotting. These experiments demonstrated that tombusviruses replicated only at a ~15% level in the absence of Rab5 proteins (Fig 1D and 1E, lanes 3–4 versus 1–2). Complementation of tombusvirus replication by expression of wt Vps21p in *vps21Δypt52Δypt53Δ* yeast supported almost wt level of viral RNA accumulation (Fig 1D and 1E, lanes 5–6), demonstrating that a single Rab5 gene provides enough proviral function for viral replication. Interestingly, expression of the active GTP-bound form Vps21Q_66_L in *vps21Δ ypt52Δ ypt53Δ* yeast also complemented the proviral function (Fig 1D and 1E, lanes 9–10), whereas expression of the GDP-bound form Vps21S_21_L could not provide the missing function (lanes 7–8). The lack of complementation was also observed when Vps21S_21_L was expressed to a high level from a high copy plasmid (S1A and S1B Fig), confirming that, unlike the active GTP-bound form Vps21Q_66_L, the GDP-bound form Vps21S_21_L cannot complement TBSV or CIRV replication in *vps21Δypt52Δypt53Δ* yeast. Expression of other small GTPases, such as the Ypt7p (Rab7 homolog, affecting late endosome to vacuole transport), Ypt6p (Rab6 homolog, involved in early endosome to Golgi retrograde transport), or Ypt32p (Rab11 homolog, mediating budding of post-Golgi vesicles from the trans-Golgi) [24], could not complement Rab5 proviral function in *vps21Δypt52Δypt53Δ* yeast (S2 Fig). Thus, Rab5 seems to have specific pro-tombusvirus function.

Similar to other GTPases, Rab5 also requires GEFs for activation, which are provided by Vps9p and Muk1p in yeast [17]. Testing of tombusvirus replication in *vps9Δmuk1Δ* yeast revealed poor repRNA replication for both TBSV and CIRV (Fig 1F and 1G, lanes 4–6 versus 1–3). Similar to the situation observed in *vps21Δypt52Δypt53Δ* yeast, the accumulation level of both replication proteins of TBSV and CIRV was reduced in *vps9Δmuk1Δ* yeast. Altogether, these data support the model that tombusviruses co-opt the active GTP-bound form of Rab5 for proviral function.

Testing the activity of the in vitro assembled tombusvirus replicase based on purified recombinant replication proteins in yeast cell-free extracts (CFEs) revealed similar activities for wt or *vps21Δypt52Δypt53Δ* CFEs (Fig 1H), including the membrane fractions (Fig 1I). The results likely reflect the differences in cell-based and cell-free assays, because the latter performs only a single full round of replication [25]. In contrast, cells support multiple rounds of tombusvirus replication during a 22 h incubation period. Therefore, we suggest that the proviral function of the co-opted Rab5 is required at the peak time (late stage) of tombusvirus replication, although it might not be limiting at the early stage of replication, which is in line with the ~15% level of replication in yeast lacking Rab5 orthologs (Fig 1D and 1E).

### Rab5 Has a Proviral Function in Plants

**Fig 2:**
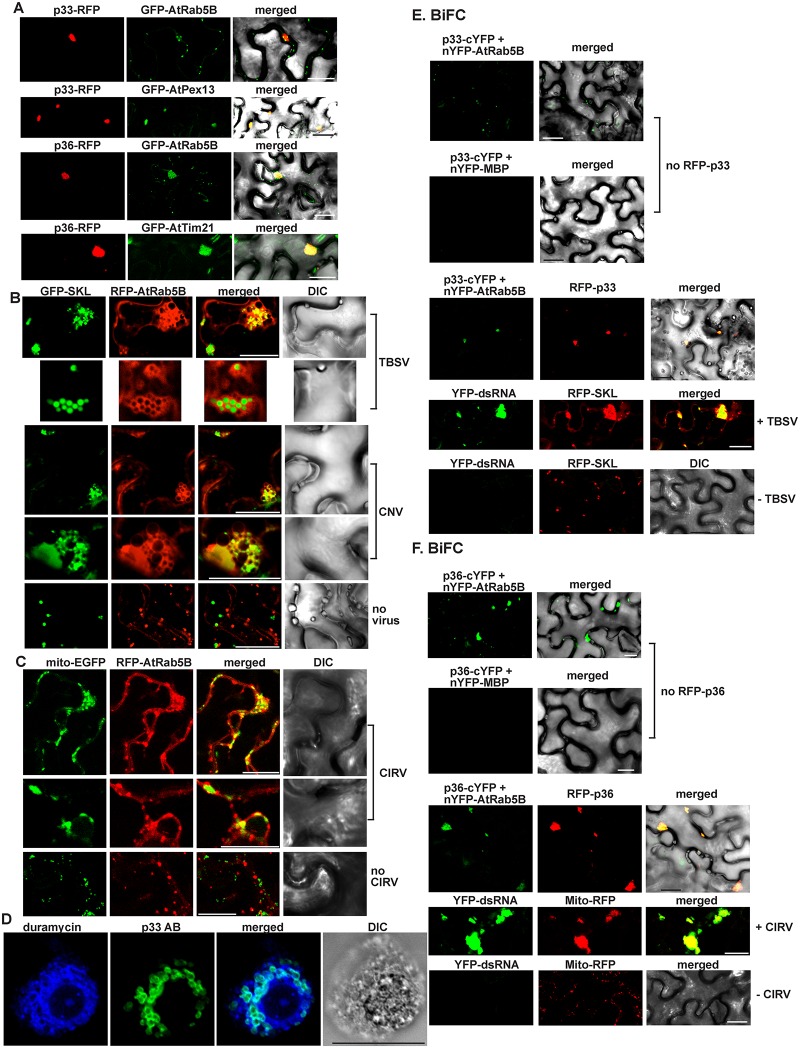
Recruitment of *Arabidopsis* Rab5 into the tombusvirus replication compartment in *N*. *benthamiana*. (A) Confocal laser microscopy shows partial co-localization of TBSV RFP-tagged p33 replication protein or CIRV RFP-tagged p36 with the GFP-AtRab5B protein in *N*. *benthamiana* cells. Expression of the above proteins from the 35S promoter was achieved after agro-infiltration into *N*. *benthamiana* leaves. Scale bars represent 20 μm. (B) Partial re-localization of RFP-AtRab5B protein to the peroxisomes (marked by GFP-SKL) in *N*. *benthamiana* cells infected with either TBSV or CNV. The bottom image shows the absence of re-localization of RFP-AtRab5B protein to the peroxisome in the mock-infected plant leaves. Scale bars represent 20 μm. (C) Partial re-localization of RFP-AtRab5B protein to the mitochondria (marked by mito-EGFP) in *N*. *benthamiana* cells infected with CIRV. The bottom image shows the absence of re-localization of RFP-AtRab5B protein to the mitochondria in the mock-infected plant leaves. Scale bars represent 20 μm. (D) TBSV infection induces membrane proliferation, which is occasionally visualized as aggregated circle-like structures. These membranous structures are enriched in PE in plant cells. The confocal laser microscopy image shows the enrichment of PE and its co-localization with the TBSV p33/p92 replication proteins, which were detected with p33-specific primary antibody and secondary antibody conjugated with Alexa Fluor488. Localization of PE is detected by using biotinylated duramycin peptide and streptavidin conjugated with Alexa Fluor 405. DIC images are shown on the right. Scale bars represent 20 μm. (E) Top image: *In planta* interaction between TBSV p33-cYFP replication protein and the nYFP-AtRab5B protein. Expression of the above proteins from the 35S promoter was done after agro-infiltration into *N*. *benthamiana* leaves. Note that p33-cYFP and the nYFP-AtRab5B protein were detected by bimolecular fluorescence complementation (BiFC). Control BiFC experiments included nYFP-MBP protein. Bottom images: The interaction between p33 replication protein and AtRab5B occurs in the replication compartment decorated by RFP-p33. As expected, the enlarged replication compartment (highlighted via RFP-SKL) also contained the viral dsRNA replication intermediate only in TBSV-infected cells (second panel form the bottom) but not in the mock-inoculated cells (bottom panel). Scale bars represent 20 μm. (F) The corresponding experiments with the CIRV p36 protein and AtRab5B (see panel E for details). Scale bars represent 20 μm.

To test if Rab5 is co-localized with the tombusvirus replication proteins in plant cells, we co-expressed them in *Nicotiana benthamiana* leaves. Interestingly, the green fluorescent protein (GFP)-tagged *Arabidopsis* Rab5B is partly co-localized with both the TBSV p33 (Fig 2A top panels and S1 Video) and the CIRV p36 (Fig 2A, bottom panels), which are known to form large viral replication compartments in the peroxisomal boundary membrane and the mitochondrial outer membranes, respectively (Fig 2A) [26,27]. These experiments confirmed that the expression of either TBSV p33 or CIRV p36 is enough to recruit Rab5 into the replication compartment in the absence of additional viral components.

To study if the recruitment of Rab5 into the replication compartment is comparable in case of a full TBSV infection of *N*. *benthamiana* plants, we observed the subcellular distribution of RFP-AtRab5B by confocal microscopy. Interestingly, we found that AtRab5B was intensely co-localized with the peroxisomal marker (GFP-SKL, matrix targeted), which showed the characteristic TBSV-induced aggregated forms of peroxisomes (Fig 2B, top). AtRab5-containing membranes frequently formed circles around the peroxisomal matrix (Fig 2B, second panel from the top). Similar structures, including peroxisome-localized Rab5B-containing membranes, were visible when *N*. *benthamiana* plants were infected with Cucumber necrosis virus (CNV, a very close relative of TBSV) (Fig 2B). As expected, uninfected *N*. *benthamiana* plants did not show co-localization of the peroxisomal GFP-SKL and the early endosomal AtRab5b and also lacked aggregated peroxisomes (Fig 2B, the lowest panel). CIRV-infected *N*. *benthamiana* plants showed the intensive co-localization of AtRab5B and the mitochondrial marker (mito-EGFP) (Fig 2C), including areas showing the characteristic CIRV-induced aggregated mitochondria. Uninfected *N*. *benthamiana* plants did not show localization of AtRab5b to the mitochondria and also lacked aggregated mitochondria (Fig 2C, the lowest panel). Thus, all these *in planta* experiments showed the robust recruitment of AtRab5B into the viral replication compartments. In addition, the aggregated round-shaped membranous structures visible in TBSV or CNV-infected plant cells (Fig 2B) are similar to those detected when p33 is visualized with antibody and PE is detected with biotinylated duramycin (which specifically interacts with PE, Fig 2D), suggesting that these structures are extensive virus-induced membrane deformations that contain co-opted Rab5. The enrichment of PE at replication sites during TBSV or CIRV replication is based on a specific process and is not due to condensation of membranes [12]. Indeed, PC detected by fluorescently labeled monoclonal antibody was not enriched at replication sites during TBSV or CIRV replication (S3 Fig).

To confirm that plant Rab5 is recruited into the viral replication compartment via the interaction with the p33 or p36 replication proteins, we have performed bimolecular fluorescence complementation (BiFC) experiments in *N*. *benthamiana* leaves. The confocal microscopic images revealed the localization of p33-AtRab5B complex (detected via BiFC) in the replication compartment (detected via RFP-p33) (Fig 2E). As expected, the large replication compartments contained the viral dsRNA and the peroxisomal marker (RFP-SKL) as well (Fig 2E, lower panels). We have obtained comparable BiFC results with the CIRV p36-AtRab5B complex, which is present in the replication compartments formed at the mitochondria (Fig 2F). Pull-down experiments with purified recombinant replication proteins further supported direct interaction of TBSV p33 and CIRV p36 with AtRab5B (Fig 1C, lanes 5–6). Altogether, the data from yeast and plant cells strongly support the model that Rab5 is recruited by the viral replication proteins into the viral replication compartments in peroxisomes and mitochondria, respectively, during TBSV and CIRV replication.

### Rab5 Is Required for the Enrichment of PE in the Viral Replication Compartment in Yeast and Plants

To test the proviral functions of Rab5 during tombusvirus replication, we measured the half-life of the p33 and p92^pol^ replication proteins, which accumulated to only low levels in *vps21Δypt52Δypt53Δ* (Fig 1D) or *vps9Δmuk1Δ* yeasts (Fig 1F). These experiments revealed significantly reduced half-life of p33 and p92^pol^ replication proteins in *vps21Δypt52Δypt53Δ* yeast in comparison with the wt yeast (S4 Fig).

**Fig 3:**
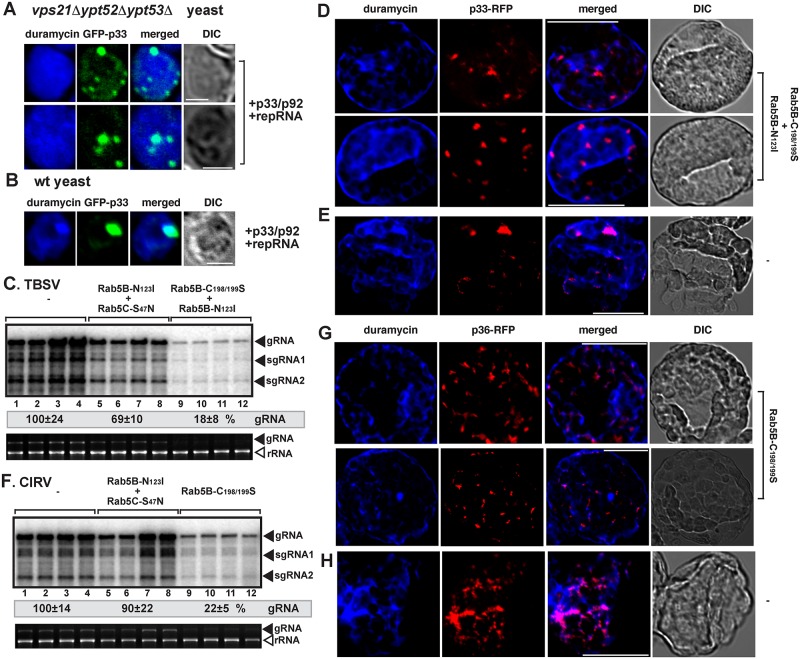
Rab5 is required for the tombusvirus-driven PE enrichment in the tombusvirus replication compartment in yeast and plants. (A) Confocal laser microscopy images show the lack of enrichment of PE at peroxisomal sites of TBSV GFP-p33 accumulation in *vps21Δypt52Δypt53Δ* yeast. (B) PE enrichment at replication sites in wt yeast. See further details in Fig 2D. Scale bars represent 2 μm. (C) Inhibition of TBSV RNA accumulation in plants by co-expression of two dominant negative mutants of AtRab5 proteins. Co-expression of the AtRab5 proteins was done from the 35S promoter in *N*. *benthamiana* leaves, which were co-infiltrated with *Agrobacterium*, and then sap-inoculated with TBSV at 1.75 dpi. The control samples were obtained from leaves expressing no AtRab5 proteins (lanes 1–4). Total RNA was extracted from leaves 3 d after sap-inoculation with TBSV. The accumulation of TBSV RNAs in *N*. *benthamiana* leaves was analyzed by northern blot. The rRNA, visualized by ethidium-bromide staining, was used as a loading control. Each experiment was performed three times. (D) Lack of PE enrichment at TBSV replication sites in plant cells co-expression of two dominant negative mutants of AtRab5 proteins. Confocal laser microscopy images show PE localization is different than the subcellular locations of the RFP-tagged TBSV p33 expressed from *35S* promoter. DIC images are shown on the right. Scale bars represent 20 μm. (E) The image shows the partial co-localization and enrichment of PE in the p33-RFP decorated replication sites in the absence of the two dominant negative mutants of AtRab5 proteins. Scale bars represent 20 μm. (F) Inhibition of CIRV RNA accumulation in plants by co-expression of two dominant negative mutants of AtRab5 proteins. CIRV infection was launched via agro-infiltration 1 d after expression of AtRab5 mutants via agro-infiltration. See further details in panel C. (G) Lack of PE enrichment at the mitochondrial CIRV replication sites in plant cells co-expression of two dominant negative mutants of AtRab5 proteins. See further details in panel D. Scale bars represent 20 μm. (H) A confocal microscopy image shows the partial co-localization and enrichment of PE in the CIRV p36-RFP decorated replication sites in the absence of the two dominant negative mutants of AtRab5 proteins. Scale bars represent 20 μm.

We previously observed decreased stability for p33 and p92^pol^ replication proteins in yeasts defective in PE synthesis [12]. Therefore, we have tested if PE is enriched in the tombusvirus replication compartments in *vps21Δypt52Δypt53Δ* yeast. Interestingly, in comparison with wt yeast, the distribution of PE changed dramatically in *vps21Δypt52Δypt53Δ* yeast, and PE was not enriched in the viral replication compartment, as visualized by the GFP-p33 decorated large punctate structures (Fig 3A versus 3B and S5 Fig). This indicated that Rab5 might be involved in enrichment of PE in the replication compartment.

To study if the plant Rab5 might play similar role in tombusvirus-driven PE enrichment in the replication compartment, we expressed dominant negative AtRab5B and Rab5C mutants in *N*. *benthamiana* leaves replicating TBSV (Fig 3C). Expression of various combinations of Rab5 mutants inhibited TBSV RNA accumulation by up to 80%, demonstrating that Rab5 is also critical for TBSV replication in plants. Unlike in control leaves, in which p33 was localized to areas enriched for PE (Fig 3E and S6B Fig) [12], plant cells expressing dominant negative AtRab5 mutants did not show PE enrichment in p33-containing areas (Fig 3D and S6A Fig). Comparable results were obtained with CIRV. Indeed, expression of dominant negative Rab5B mutant inhibited CIRV replication by ~80% (Fig 3F) and interfered with the enrichment of PE at the mitochondrial replication compartments (Fig 3G versus 3H and S6C and S6D Fig). Thus, these data highlighted the involvement of Rab5 in PE enrichment at either peroxisomal or mitochondrial replication sites.

**Fig 4:**
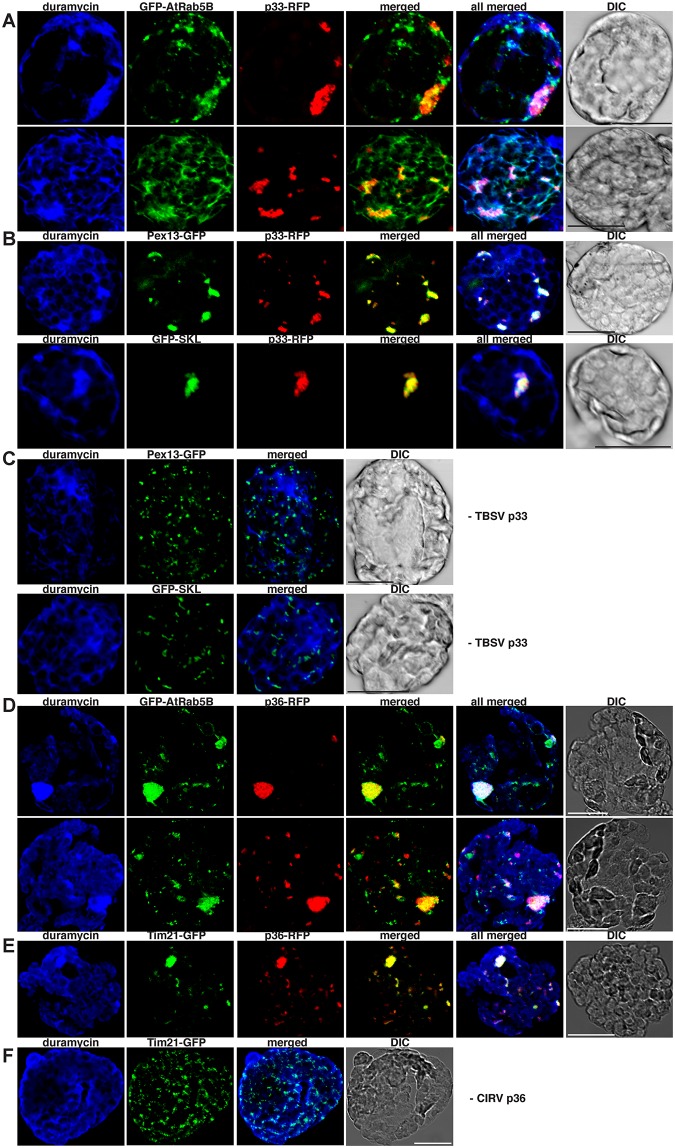
Rab5 is partly co-localized with PE-enriched tombusvirus replication compartment in plant cells. (A) Confocal laser microscopy images show the co-localization of GFP-AtRab5B with the TBSV p33-RFP replication protein in subcellular areas enriched with PE in *N*. *benthamiana* protoplasts. Scale bars represent 20 μm in each panel. (B) Confocal laser microscopy images confirm that these subcellular areas are derived from aggregated peroxisomes based on co-localization with either Pex13-GFP peroxisomal membrane marker protein or GFP-SKL peroxisomal luminal marker protein. (C) Control images show the lack of PE enrichment in peroxisomes in the absence of viral components. Note the absence of aggregated peroxisomes in these cells. (D) Confocal laser microscopy images show the co-localization of GFP-AtRab5B with the CIRV p36-RFP replication protein in subcellular areas enriched with PE. (E) Confocal laser microscopy images confirm that these subcellular areas are derived from aggregated mitochondria based on co-localization with Tim21-GFP marker protein. (F) Control images show the lack of PE enrichment in mitochondria in the absence of viral components. Note the absence of aggregated mitochondria in these cells.

To examine if Rab5 is localized in PE-enriched compartments in the presence of tombusvirus replication proteins, we co-expressed GFP-AtRab5B with p33-RFP and detected PE in the same plant cells. We found that the TBSV replication compartment decorated by p33-RFP showed highly enriched PE content and co-localized with AtRab5B (Fig 4A and S7A Fig) and peroxisomal marker (GFP-SKL or AtPex13-GFP) (Fig 4B and S7B Fig). As expected, the peroxisomal marker was not localized in PE-rich areas in the absence of viral replication proteins (Fig 4C and S7C Fig). Comparable results were obtained with CIRV p36 replication protein, which co-localized with GFP-AtRab5B (Fig 4D and S7D Fig) and the AtTim21-GFP mitochondrial marker (Fig 4E and S7E Fig) within highly PE-enriched compartments, seen as large punctate structures. The AtTim21-GFP mitochondrial marker was not localized in PE-rich areas in the absence of viral replication protein (Fig 4F and S7F Fig). These results are in agreement that a major proviral function of the co-opted Rab5 is to facilitate PE enrichment within the replication compartments regardless of whether those are formed in the aggregated peroxisomal or mitochondrial membranes.

**Fig 5:**
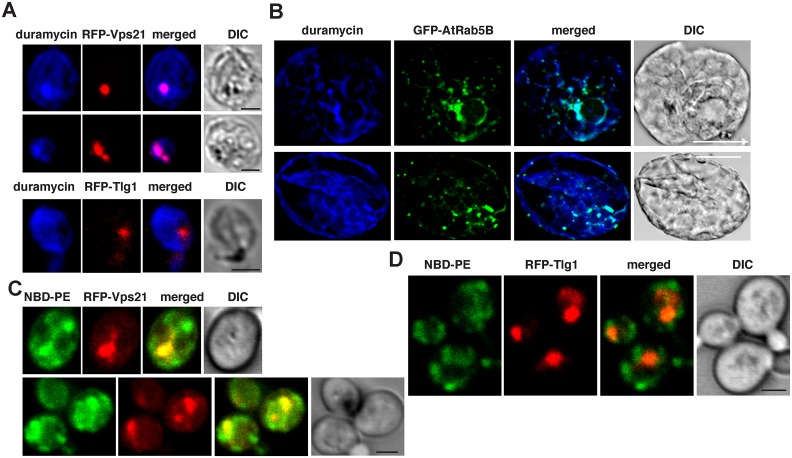
The early endosomal membranes are enriched with PE in yeast and plant cells. (A) Confocal laser microscopy images show the enrichment of PE and its co-localization with the early endosomal RFP-tagged Vps21p expressed from *TEF1* promoter in the absence of tombusviral components in wt yeast cells (top two images). DIC images are shown on the right. Localization of PE is detected by using biotinylated duramycin peptide and streptavidin conjugated with Alexa Fluor 405. The bottom image shows the lack of PE enrichment in trans-Golgi network marked by RFP-Tlg1. Scale bars represent 2 μm. (B) Confocal laser microscopy images show the enrichment of PE with the endosomal GFP-tagged AtRab5B expressed from 35S promoter in the absence of tombusviral components in *N*. *benthamiana* cells. Scale bars represent 20 μm. (C) Enrichment of exogenous PE in early endosomes labeled with RFP-Vps21 protein in wt yeast cells. Yeast cells were cultured (initial 0.3 OD_600_) with 80 μM NBD-PE for 12–14 h. Scale bars represent 2 μm. (D) The control panel shows minimal level of NBD-PE enrichment in the trans-Golgi network labeled with RFP-Tlg1 in wt yeast cells. Scale bars represent 2 μm.

Because the membrane of the early (Rab5-positive) endosome is rich in PE [28], recruitment of the GTP-bound Rab5 by tombusviruses might lead to enrichment of PE in the viral replication compartment. To test if PE level is high in the Rab5-positive endosomes in yeast and plant cells, we examined the subcellular distribution of PE with RFP-Vps21 in yeast and AtRab5B in plant cells lacking viral components. Interestingly, Vps21 (Fig 5A and S8A Fig) and AtRab5B (Fig 5B and S8B Fig) were localized in PE-enriched membranes in yeast and plant cells. Because Vps21 and Rab5B are early endosomal proteins, these data support the concept that the Rab5-positive endosomal membranes are rich in PE, although some other subcellular membranes are also rich in PE based on PE distribution in the virus-free yeast and plant cells (Fig 5A and 5B and S8A and S8B Fig). The trans-Golgi network marker, Tlg1p (Fig 5A, bottom panel and S8A Fig), the peroxisomal marker (Fig 4C and S7C Fig), or the mitochondrial marker (Fig 4F and S7F Fig), however, did not co-localize with PE-rich membranes, suggesting that PE is not distributed evenly within the cellular endomembrane and organellar systems.

Addition of the fluorescent NBD-PE to the yeast culture media resulted in the highest enrichment of NBD-PE in the Rab5-positive endosomes (see comparison with RFP-Vps21 endosomal marker) (Fig 5C and S8C Fig), while NBD-PE was not enriched in the Trans-Golgi network based on the Tlg1 marker protein (Fig 5D and S8D Fig). Thus, these data also support that the Rab5-positive endosomal membranes have high PE content in the absence of tombusviral components.

**Fig 6:**
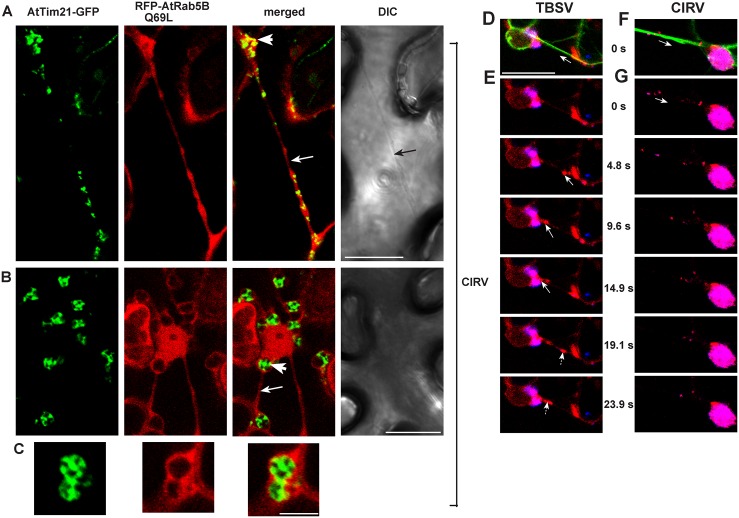
The role of actin filaments in recruitment of Rab5-positive endosomes to the large tombusviral replication compartments in plant cells. (A–B) Confocal laser microscopy images of CIRV-infected *N*. *benthamiana* cells expressing AtTim21-GFP mitochondrial marker and the RFP-tagged active GTP-locked AtRab5B mutant. Note the large aggregated mitochondria-containing area (marked by a white arrowhead) and the actin-like filamentous structure (pointed at by a white arrow). Scale bars represent 20 μm. (C) An enlarged subcellular area showing the aggregated mitochondria and the RFP-AtRab5 mutant. Scale bar represents 5 μm. (D–G) Still images from a movie taken from plant cells co-expressing RFP-AtRab5B with TBSV p33-BFP (D–E) or CIRV p36-BFP (F–G) in transgenic plants expressing GFP-mTalin (an actin filament marker). Scale bars represent 20 μm. (D and F) All three channels from 0s are shown. White arrow depicts the direction of Rab5-positive endosomes (red) moving towards the replication compartment (blue) via actin filaments (green). (E and G) Merged images of RFP-AtRab5B and p33-BFP/p36-BFP. White arrow shows the movement of Rab5-positive endosomes. Scale bars represent 20 μm. See S2 and S3 Videos.

The enrichment of the endogenous PE or the exogenous NBD-PE in the tombusvirus-induced replication compartment [12] versus their high-level, Rab5-positive endosomal distribution in the absence of viral components (Figs 4C and 4F and 5) suggests that tombusviruses recruit the Rab5-positive endosomes to the sites of replication. Accordingly, this is supported by the direct interaction between the viral replication proteins and the active GTP-Rab5 (Fig 1), which is localized to the endosomal membranes via its prenylated C-terminus [16]. Moreover, expression of AtRab5B-Q_69_L GTP-locked active form shows co-localization with the mitochondrial marker AtTim21 in plant cells infected with CIRV (Fig 6A and 6B). To obtain further evidence of the recruitment of Rab5-positive endosomes to the sites of tombusvirus replication, we observed the distribution of the low abundant phosphatidylinositol 3-phosphate (PI3P) lipid, which is a well-characterized endocytic compartment marker [29]. We found partial co-localization of p33-decorated viral replication compartments with PI3P (detected via the GFP-2xFYVE PI3P sensor) in *N*. *benthamiana* cells (S9 Fig). As expected, PI3P co-localized with Rab5B but not with the peroxisomal marker in plant cells lacking viral components (S9B and S9C Fig). We confirmed the partial recruitment of PI3P to the mitochondrial replication compartment in the presence of CIRV p36 replication protein (S9D Fig). Altogether, these data further support the recruitment of a significant portion of Rab5-positive endosomes to the tombusvirus replication compartments.

PE is synthesized by phosphatidylserine (PS) decarboxylase (Psd1p or Psd2p, involved in de novo synthesis of PE from PS) or by using ethanolamine by Ept1p and Cpt1p [30]. Among these enzymes, only Psd2p is present in the endosomal membranes. However, deletion of *PSD2* does not affect TBSV replication in yeast, likely due to the complementing effects of the other PE-producing enzymes/pathways (S10 Fig). To test in more detail if deletion of the PE-synthesizing enzymes/pathways could affect TBSV replication, we grew *psd1Δ* and *psd2Δ* yeasts without ethanolamine in the culture media [31]. We found that TBSV repRNA accumulation was two times higher in *psd1Δ* yeast than in *psd2Δ* yeast (S10 Fig lanes 5–8 versus 9–12). Similarly, *psd1Δept1Δcpt1Δ* yeast (cultured without ethanolamine), which could produce PE only via the endosomal Psd2p, supported TBSV replication by ~2.5x higher efficiency than the control yeast strains (S10 Fig lanes 17–20). Therefore, TBSV might be able to recruit PE more efficiently when PE is only synthesized in the endosomes by Psd2, although this enzyme is not fully required, likely due to the rapid transfer of PE in the cells via the endosomal pathway from other subcellular compartments (see also the NBD-PE distribution, Fig 5C).

### The Role of the Actin Network in Recruitment of Rab5-Positive Endosomes into the Viral Replication Compartment

The above data support the recruitment of Rab5-positive endosomes to the replication compartment. But how can the tombusvirus recruit the endosomes to aggregated peroxisomes (in case of TBSV) or aggregated mitochondria (in case of CIRV)? Interestingly, co-expression of AtRab5B-Q_69_L GTP-locked active form with the mitochondrial marker AtTim21 in plant cells infected with CIRV frequently visualized actin-like filamentous distribution (Fig 6A and 6B). To test if the actin filaments play roles in recruitment of Rab5-positive endosomes to the replication compartments, we used transgenic *N*. *benthamiana* plants expressing GFP-tagged mTalin, which binds to actin filaments [32]. Video images of plant cells (S2 and S3 Videos) expressing either TBSV or CIRV BFP-tagged replication proteins show the rapid recruitment of the RFP-AtRab5B decorated endosomes along the actin cables into the peroxisomal (Fig 6D and 6E and S2 Video) or mitochondrial (Fig 6F and 6G and S3 Video) replication compartments. These data are in agreement with the model that tombusviruses actively recruit Rab5-positive endosomes into the replication compartments via using the stable actin filaments.

## Discussion

(+)RNA viruses build large replication compartments or organelles during the infection process to support viral replication in a protected subcellular environment [33–36]. Although building of the replication compartments is driven by viral replication proteins, viruses have to co-opt an unknown number of host proteins, cellular membranes, and lipids, and even alter cellular metabolism to accomplish these large and intricate subcellular structures. Tombusviruses and yeast serve as simple model systems to identify the host factors involved and to dissect the mechanisms required for building the viral replication compartments. The use of two different tombusviruses, TBSV utilizing primarily the peroxisomal membranes while CIRV usurps the mitochondrial outer membranes, allows for studying the similarities in building the replication compartments in different subcellular niches.

In this work, we have found a major role for Rab5 small GTPase in the formation of tombusvirus replication compartment. The observed direct interaction between the viral replication proteins (either TBSV p33 or CIRV p36) and the active GTP-bound form of Rab5 allows tombusviruses to co-opt Rab5 to the replication compartments, which is required for robust replication. Accordingly, we observed little tombusvirus replication in the absence of the three Rab5 orthologs in yeast (Fig 1) or when dominant negative forms of plant Rab5 were expressed in *N*. *benthamiana* (Fig 3). Co-opting of the endosome-localized GTP-bound Rab5 by tombusviruses facilitates the recruitment of endosomes, as indicated by capturing of the endosomal PI3P lipid in the replication compartment (S9 Fig). Although we have not characterized this in detail, PI3P might have a role in TBSV replication. Furthermore, the endosomal compartment contains ESCRT proteins/complexes, which might also facilitate their recruitment into TBSV replication [37]. The endosomal membrane also contains Psd2p PS decarboxylase involved in de novo synthesis of PE from PS [30], which could possibly further enrich PE in the replication compartment. Indeed, increased PE synthesis has been shown in yeast or plants replicating tombusviruses [12]. Altogether, the tombusviral replication organelles have similar components with Rab5-positive endosomes, including high PE content, ESCRT proteins, Rab5 GTPase, and PI3P. Moreover, the TBSV replication organelles show multivesicular body (MVB)-like structures, which are morphologically similar to late endosomes/MVBs formed from Rab5-positive endosomes [38,39].

The presented data reveal that one major benefit for tombusviruses to recruit the Rab5-positive endosomes via replication protein–Rab5 interaction is the surplus of PE-rich endosomal membrane utilized to build the large PE-enriched replication compartment. Accordingly, in vitro, yeast-based and plant works have shown the great dependence of tombusviruses on high enrichment of PE in VRCs [12]. The high PE level likely facilitates the stable formation of spherules due to the known feature of PE to induce negative membrane curvature [40,41] and the activation of the tombusvirus RdRp [13].

**Fig 7:**
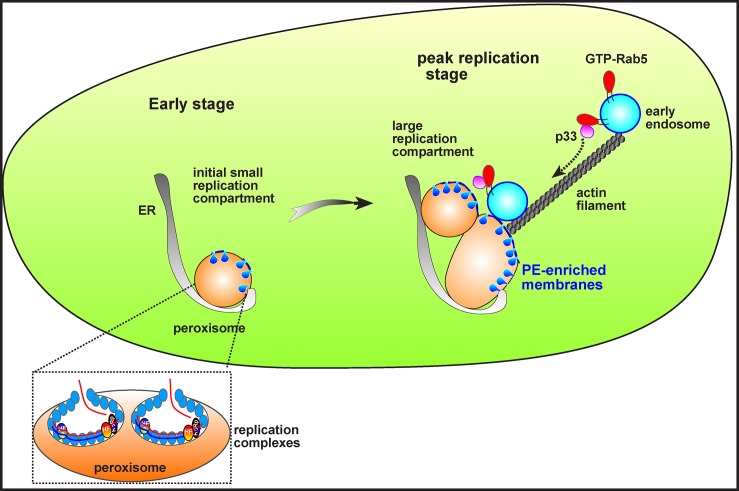
A model on the roles of p33-mediated recruitment of Rab5-positive endosomes in the formation of large tombusviral replication compartments. At the early stage of tombusvirus replication, TBSV-induced spherule formation may take place in the existing peroxisomal membranes. At the peak time of replication, occurring at a late stage at which point the viral components are much more abundant due to ongoing translation of viral RNAs, however, a portion of p33 molecules co-opt the PE-rich Rab5-positive endosomes via p33–GTP-Rab5 interaction using the actin cables. These processes lead to the formation of large replication compartments containing aggregated peroxisomes fused with PE-rich Rab5-positive endosomes, providing the suitable microenvironment for building numerous spherules harboring the active tombusvirus VRCs. We envision similar early and peak/late stages with CIRV, except the involvement of mitochondria in building the viral replication compartment.

Our results also reveal that both the peroxisomal TBSV and the mitochondrial CIRV use similar strategies with high efficiency to hijack the Rab5-positive endosomes into the viral replication compartments. Tombusviruses utilize the actin network to accomplish this feat in plant cells (Fig 6 and S2 and S3 Videos). It is known that tombusviruses manipulate the actin network via blocking the function of Cof1p (cofilin/ADF), which is an actin filament depolymerizing factor [42]. Due to the p33-driven inhibition of Cof1p activity [32,43], the actin filaments are stabilized during tombusvirus replication, facilitating the efficient transport of cargoes to the replication compartments. Accordingly, the large replication compartments are located at the crossroads of actin filaments [32]. Based on these observations, we propose that one of the major functions of the (virus-mediated) stabilized actin filaments is to deliver the Rab5-positive endosomes captured via replication protein–GTP-Rab5 interaction to the sites of replication to build the large and protective replication compartment that is initially formed on either peroxisomal (in case of TBSV) or mitochondrial membranes (in case of CIRV). Thus, the replication protein–GTP-Rab5 interaction might be needed mostly during the peak replication period that coincides with the appearance of large replication compartments [44]. Indeed, the role of Rab5 was minimal during cell-free replication, which consists of only one full cycle of RNA synthesis (Fig 1). Altogether, our current model predicts that, after the assembly of a limited number of tombusvirus VRCs at peroxisomal or mitochondrial membranes to support initial replication, tombusviruses recruit the Rab5-positive endosomes to channel large amount of PE-rich membranes for new VRC assembly, leading to the formation of MVB-like replication organelles at the latter stage of replication. Thus, hijacking Rab5-positive endosomes is critical for tombusvirus replication to reach to peak level by providing additional PE-rich membranes and possibly other host factors (Fig 7).

It is known that other (+)RNA viruses co-opt small cytosolic GTPases to boost virus replication. For example, enteroviruses co-opt Arf1 small GTPase and its guanine nucleotide exchange factor GBF1 to recruit downstream effectors [45,46]. One of these effectors is phosphatidylinositol-4-kinase IIIbeta, which generates PI4P-rich membranes that are needed for recruitment of the viral RdRp and the formation of the viral replication organelle. Some enteroviruses also co-opt the recycling endosomal compartment through Rab11 small GTPase to enrich sterols in the viral replication organelles [47].

RNAi-based down-regulation of Rab5A and Rab7L1 inhibited Hepatitis C virus (HCV) replication, suggesting that these small GTPases are host factors for HCV [48]. Moreover, HCV recruits Rab5 and Rab7 into foci containing the NS4B replication protein, which remodels host membranes to form HCV replication complexes. Expression of dominant negative mutant of Rab5 inhibited the NS4B foci formation [49]. HCV replication also depends on Rab27a, which controls membrane trafficking, microvesicle transport pathways [50], and Rab1, which is recruited to lipid droplets by the NS5A replication protein to promote viral replication [51]. Similarly, Dengue virus (DENV) takes advantage of Rab18, which facilitates the recruitment of fatty acid synthase and NS3 replication protein to lipid droplets, probably to facilitate sufficient lipid supply for membrane proliferation needed for DENV replication [52].

Although the proviral functions of plant Rab small GTPases have not yet been defined in detail, the role of the Golgi-localized NbRABG3f is important for *Bamboo mosaic virus* replication [53]. Also, Arf1 small GTPase binds to the p27 replication protein of *Red clover necrotic mosaic virus*, and inhibition of Arf1 activity disrupted VRC assembly [54]. Small GTPases of the secretory pathway also affect the replication of potyviruses [55]. Thus, the emerging view is that replication of both animal and plant RNA viruses greatly depends on a subset of small GTPases of the secretory/endocytic pathways, likely offering targets for future antiviral interventions.

### Summary

In this work, we demonstrate that tombusviruses subvert Rab5-positive endosomes to build complex replication compartments involving either peroxisomes or mitochondria. These events supply the proviral PE to facilitate the membranous microenvironment during peak-level viral replication. This hijacked cellular pathway for supporting PE enrichment in the viral replication compartment might provide an intriguing target for disease control.

## Material and Methods

### Yeast Strains and Expression Plasmids

The yeast strains and plasmids and their constructions are described in S1 Text.

### Analysis of TBSV or CIRV repRNA Replication in Yeast

To measure the effects of deletion of specific host genes on replication of TBSV or CIRV, yeast strains BY4741, *vps21Δypt52Δypt53Δ*, and *vps9Δmuk1Δ*, respectively, were transformed with plasmids pESC-T33/DI72 and pYES-T92 for TBSV replication [56] or pESC-C36/DI1 and pYES-C95 for CIRV replication [57]. Tombusvirus repRNA replication was induced by culturing cells in synthetic complete medium lacking urea, leucine, and histidine, with 2% galactose medium after overnight culture in synthetic complete medium lacking urea, leucine, and histidine with 2% glucose, and then yeast was grown for 22 h at 23°C.

For overexpression and complementation studies, plasmids expressing Vps21 and its mutants (low copy number plasmids pRS315-pCUP1-Flag-Vps21, pRS315-pCUP1-Flag-Vps21-S21L, or pRS315-pCUP1-Flag-Vps21-Q66L) were transformed into yeast strains BY4741 or *vps21Δypt52Δypt53Δ*, together with plasmids expressing viral components (see above). Culturing condition for yeast was performed as described above, except that 50 μM copper sulfate was added into in synthetic complete medium lacking urea, leucine, and histidine, with 2% galactose medium after overnight culture to induce Flag-tagged Vps21 expression.

Total RNA was extracted from yeast cells and subjected for repRNA detection using northern blotting, as described previously [58]. Total proteins were extracted to detect 6xHis-tagged tombusvirus replication proteins and Flag-tagged Vps21 or its mutants expression using western blotting and primary anti-His antibody and anti-Flag antibody, respectively, followed by secondary anti-mouse antibodies conjugated with alkaline phosphatase.

### Confocal Microscopic Analysis of Plant Epidermal Cells

To analyze the subcellular localization of host proteins in the presence or absence of viral components in *N*. *benthamiana* leaves, agro-infiltration was used to express recombinant proteins tagged with either GFP or RFP from 35S promoter using *Agrobacterium* cultures (OD_600_ value of 0.5). Infiltrated leaves were harvested at 2 dpi, and they were immediately subjected to confocal microscopic analysis using 488-nm laser for GFP, 543-nm for RFP, or 405-nm for BFP in an Olympus FV1000 confocal laser scanning microscope. The movies were created by OLYMPUS FLUOVIEW software.

Viral double strand RNA (dsRNA) generated during tombusvirus replication was visualized by a dsRNA binding-dependent fluorescence complementation assay [59]. The dsRNA sensor YN-B2 and YC-VP35 plasmids were agro-infiltrated into *N*. *benthamiana* leaves at OD_600_ of 0.15, respectively, together with either RFP-SKL (peroxisome marker) or mito-RFP (mitochondria marker) at OD_600_ of 0.5. TBSV was sap-inoculated 20 min after agro-infiltration of dsRNA sensor and RFP-SKL, while CIRV infection was initiated via agro-infiltration (OD_600_ of 0.15) together with dsRNA sensor and mito-RFP. Leaves were harvested and then immediately subjected to confocal microscopic analysis 2 d after agro-infiltration. The fluorescence complementation was detected via the GFP channel (excitation/emission: 488 nm/500–530 nm).

To detect interaction of AtRab5B with TBSV p33 or CIRV p36 replication proteins using fluorescence complementation assay, pGD-nYFP-AtRab5B (or pGD-nYFP-MBP as a negative control) was agro-infiltrated with pGD-p33-cYFP or pGD-p36-cYFP (OD_600_ 0.4, each). To detect subcellular location of the interaction, pGD-T33-RFP or pGD-C36-RFP expressing RFP-tagged TBSV p33 or CIRV p36 were coagro-infiltrated (OD_600_ 0.4, each) with the above combinations. Infiltrated leaves were harvested and then subjected to confocal microscopic analysis 2 d after agro-infiltration. The fluorescence complementation was detected using the GFP channel.

### Protoplasts Preparation from Plant Leaves and Confocal Microscopic Analysis of PE and PC Distribution

Proteins were transiently expressed in *N*. *benthamiana* leaves using agro-bacterium–mediated transient expression. Infiltrated leaves were harvested at 2 dpi and used for releasing of mesophyll protoplasts based on the Sheen lab's method [60], with minor modifications. Briefly, leaves were sliced into 0.5–1 mm strips and digested with 1.5% (w/v) Cellulase and 0.4% Macerozyme in Protoplast Incubation Medium (PIM) (1 L of medium contains 4.4 g Murashige-Skoog Salts, 34.2 g sucrose, 0.58 g MES, 90.1 g mannitol, and 0.56 g CaCl_2_, pH 5.8). Digested protoplasts were passed through sieve set (Scienceware Mini-Sieve Microsieve Set from Fisher cat# 14-306A). Collected protoplasts were pelleted by centrifugation at 120x g for 10 min and washed once with 0.5 M mannitol buffer (0.5 M mannitol, 15 mM MgCl_2_, and 4 mM MES, pH 5.7), once with W5 solution (154 mM NaCl, 125 mM CaCl_2_, 5 mM KCl, 2 mM MES pH5.7), and re-suspended in 0.5 M mannitol buffer. Then, 0.55 M sucrose was layered under the 0.5 M mannitol solution with protoplast and centrifuged at 160x g for 10 min. Digested protoplast were recovered from the interface between 0.55 M sucrose and 0.5 M mannitol layers. Recovered protoplasts were pelleted and resuspended into 0.5 M mannitol buffer and subjected to PE staining and confocal microscopic analysis according to the methods described previously [12]. PE imaging for plant or yeast cells were performed as described previously [12]. PC imaging was similar to PE imaging, except that PC was detected using a monoclonal anti-PC antibody (JE-1) [61] and anti-mouse secondary antibody conjugated with Alexa Fluor 488. ImageJ software was used to show the distribution of PE and of PC in the replication compartment.

## Supporting Information

S1 Fig Complementation of tombusvirus replication by Vps21 in yeast lacking the three Rab5 orthologous genes. **(A)** TBSV repRNA accumulation is measured in *vps21Δypt52Δypt53Δ* yeast expressing His_6_-p33 and His_6_-p92 from the galactose-inducible *GAL1* promoter, and DI-72(+) repRNA from the galactose-inducible *GAL10* promoter. FLAG-tagged Vps21 or its mutants were expressed from the copper-inducible *CUP1* promoter based on high copy number plasmids. The yeast cells were pre-cultured for 12 hours at 29°C in 2% glucose SC minimal media, and then for 22 h at 23°C in 2% galactose SC minimal media supplemented with 50 μM CuSO_4_. Northern blot analysis was used to detect DI-72(+) repRNA accumulation. The accumulation level of DI-72(+) repRNA was normalized based on 18S rRNA levels (second panel from top). Bottom panels: Western blot analysis of the accumulation level of His_6_-tagged p33, His_6_-p92 and FLAG-Vps21 proteins using anti-His and anti-FLAG antibodies, respectively. Note that FLAG-Vps21 forms a double band due to prenylation (a lipidation type of posttranslational modification) that is required for binding to the endosomal membrane. The faster migrating band represents the prenylated form of Vps21 (depicted by an arrow), while the unmodified form is depicted by an open arrowhead. The total protein samples were stained with coomassie blue. Each experiment was performed three times. **(B)** Complementation of CIRV repRNA accumulation in *vps21Δypt52Δypt53Δ* yeast expressing Vps21p or its mutants. See further details in panel A.
(TIF)

S2 Fig Lack of complementation of tombusvirus replication by various yeast Rab GTPases in yeast lacking the three Rab5 orthologous genes. TBSV repRNA accumulation is measured in *vps21Δypt52Δypt53Δ* yeast expressing His_6_-p33 and His_6_-p92 from the galactose-inducible *GAL1* promoter, and DI-72(+) repRNA from the galactose-inducible *GAL10* promoter. FLAG-tagged Vps21, Ypt6, Ypt7 and Ypt32, respectively, were expressed from the copper-inducible *CUP1* promoter based on low copy number plasmids. The yeast cells were pre-cultured for 12 hours at 29°C in 2% glucose SC minimal media, and then for 22 h at 23°C in 2% galactose SC minimal media supplemented with 50 μM CuSO_4_. Northern blot analysis was used to detect DI-72(+) repRNA accumulation. The accumulation level of DI-72(+) repRNA was normalized based on 18S rRNA levels (second panel from top). Bottom panels: Western blot analysis of the accumulation level of His_6_-tagged p33, His_6_-p92 and FLAG-Vps21, Ypt6, Ypt7 and Ypt32 proteins using anti-His and anti-FLAG antibodies, respectively. Note that FLAG-Vps21, Ypt6, and Ypt7 form a double band due to prenylation (a lipidation type of posttranslational modification) that is required for binding to the subcellular membrane. The faster migrating band represents the prenylated forms (depicted by an arrow), while the unmodified form is depicted by an open arrowhead. The total protein samples were stained with coomassie blue. Each experiment was performed three times.
(TIF)

S3 Fig Lack of PC enrichment within tombusvirus replication compartment in plant cells. **(A-B)** The TBSV or CIRV-induced replication compartments are visualized by confocal laser microscopy images. p33-RFP or p36-RFP were expressed based on Agro-infiltration of *N*. *benthamiana* leaves. PC distribution was visualized by monoclonal antibody JE-1 and secondary antibody conjugated with Alexa Fluor488. DIC (differential interference contrast) images are shown on the right. Scale bars represent 20 mm. Panels on the right: ImageJ software was used to show the lack of enrichment of PC (green line) in the replication compartment (red line).
(TIF)

S4 Fig Decreased stability of TBSV replication proteins in yeast lacking the three Rab5 orthologous genes. Expression of 6xHis-tagged p33 and 6xHis-p92 in *vps21Δypt52Δypt53Δ* and wt yeasts was repressed from the *GAL1* promoter and via the addition of 100 μg/ml cycloheximide to block new protein synthesis. The total yeast protein samples were analyzed by SDS/PAGE and Western blotting with anti-His antibody to measure the accumulation level of 6xHis-tagged p33 and 6xHis-p92 at the shown time points.
(TIF)

S5 Fig Lack of enrichment of PE at TBSV replication sites in *vps21Δypt52Δypt53Δ* yeast. **(A)** Panels on the left: ImageJ software was used to show the lack of enrichment of PE (blue line) in the replication compartment (green line). Confocal laser microscopy images on the right show PE and TBSV GFP-p33 distribution in *vps21Δypt52Δypt53Δ* yeast. **(B)** PE distribution at replication sites in wt yeast. See details in panel A and Fig 3A and 3B.
(TIF)

S6 Fig Measuring PE enrichment at TBSV replication sites in *N*. *benthamiana* cells expressing dominant negative mutants of AtRab5 proteins. **(A-D)** Panels on the left: ImageJ software was used to show the enrichment of PE (blue line) in the replication compartment (red line). Confocal laser microscopy images on the right show PE and TBSV p33-RFP and CIRV p36-RFP distribution. See further details in Fig 3D–3H.
(TIF)

S7 Fig Measuring Rab5 colocalization with PE enriched TBSV replication compartment in *N*. *benthamiana* cells. **(A-F)** Panels on the left: ImageJ software was used to show the enrichment of PE (blue line), AtRab5 (green line) in the replication compartment (red line). Confocal laser microscopy images on the right show GFP-AtRab5, PE detected by duramycin, and TBSV p33-RFP or CIRV p36-RFP distribution. See further details in Fig 4A–4F.
(TIF)

S8 Fig Measuring PE-richness of Rab5-positive endosomes in the absence of tombusviruses in yeast and *N*. *benthamiana* cells. **(A)** Panels on the left: ImageJ software was used to show the enrichment of PE (blue line) on Vps21 (Rab5)-positive (red line) endosomal membranes in yeast. Confocal laser microscopy images on the right show RFP-Vps21 (or RFP-Tlg1, lower panel), and PE detected by duramycin. See further details in Fig 5A. **(B)** Panels on the left: ImageJ software was used to show the enrichment of PE (blue line) on AtRab5-positive (green line) endosomal membranes in *N*. *benthamiana* cells. Confocal laser microscopy images on the right show GFP-AtRab5 and PE detected by duramycin. See further details in Fig 5B. **(C-D)** Panels on the left: ImageJ software was used to show the enrichment of exogenous PE (green line) on Vps21 (Rab5)-positive (red line) endosomal membranes, but not in the late Golgi in yeast. Confocal laser microscopy images on the right show RFP-Vps21 (or RFP-Tlg1, panel D) and NBD-PE.
(TIF)

S9 Fig PI3P is partly co-localized with the tombusvirus replication compartment in plant cells. **(A)** Confocal laser microscopy images show the co-localization of GFP-2xFYVE (PI3P-binding motif) with the TBSV p33-RFP replication protein in subcellular areas in *N*. *benthamiana* cells. Note the large replication compartment (representing aggregated peroxisomes) in these cells. **(B)** Confocal laser microscopy images confirm the localization of PI3P with the Rab5-positive endosomes in the absence of tombusvirus proteins. **(C)** Confocal laser microscopy images confirm the separate localization of PI3P from GFP-SKL peroxisomal luminal marker protein in the absence of tombusvirus proteins. **(D)** Confocal laser microscopy images show the co-localization of GFP-2xFYVE (PI3P-binding motif) with the CIRV p36-RFP replication protein in subcellular areas in *N*. *benthamiana* cells. Note the large replication compartment (representing aggregated mitochondria) in these cells.
(TIF)

S10 Fig Altered TBSV replication in PE synthesis pathway deletion yeast strains. Replication of TBSV in yeast deletion strains grown in media without ethanolamine. Upper Panel: Northern blot of TBSV repRNA and 18S ribosomal RNA. Lower Panel: Western blot of total proteins extracted from different strains tested. TBSV p33 and p92 tagged with His6-tag were detected with an anti-HIS antibody. Total proteins were stained with Ponceau S on PVDF membrane after transfer. Each experiment was repeated.
(TIF)

S1 Text Materials and Methods. (DOCX)

S1 Data Data file. (XLSX)

S1 Video Recruitment of Arabidopsis Rab5B into the tombusvirus replication compartment in N. benthamiana. (MOV)

S2 Video Recruitment of Arabidopsis Rab5-positive endosomes into the TBSV replication compartment through actin filaments in N. benthamiana. (MOV)

S3 Video Recruitment of Arabidopsis Rab5-positive endosomes into the CIRV replication compartment through actin filaments in N. benthamiana. (MOV)

## Abbreviations

BFP: blue fluorescent protein
BiFC: bimolecular fluorescence complementation
CFEs: cell-free extracts
CIRV: Carnation Italian ringspot virus
CNV: Cucumber necrosis virus
DENV: Dengue virus
DIC: differential interference contrast
ESCRT: endosomal sorting complex required for transport
GDP: guanosine diphosphate
GEF: guanine nucleotide exchange factor
GFP: green fluorescent protein
GTP: guanosine triphosphate
HCV: Hepatitis C virus
MBP: maltose-binding protein
MVB: multivesicular body
MYTH assay: membrane yeast two-hybrid assay
PC: phosphatidylcholine
PE: phosphatidylethanolamine
PI3P: phosphatidylinositol 3-phosphate
PS: phosphatidylserine
RFP: red fluorescent protein
TBSV: Tomato bushy stunt virus
VRC: viral replication complex
wt: wild-type
