# Supplementary figures and images for: Enrichment of Phosphatidylethanolamine in Viral Replication Compartments via Co-opting the Endosomal Rab5 Small GTPase by a Positive-Strand RNA Virus
Source: PLoS Biol. 2016 Oct 19;14(10):e2000128. doi: 10.1371/journal.pbio.2000128 (PMC5070881; doi:10.1371/journal.pbio.2000128)

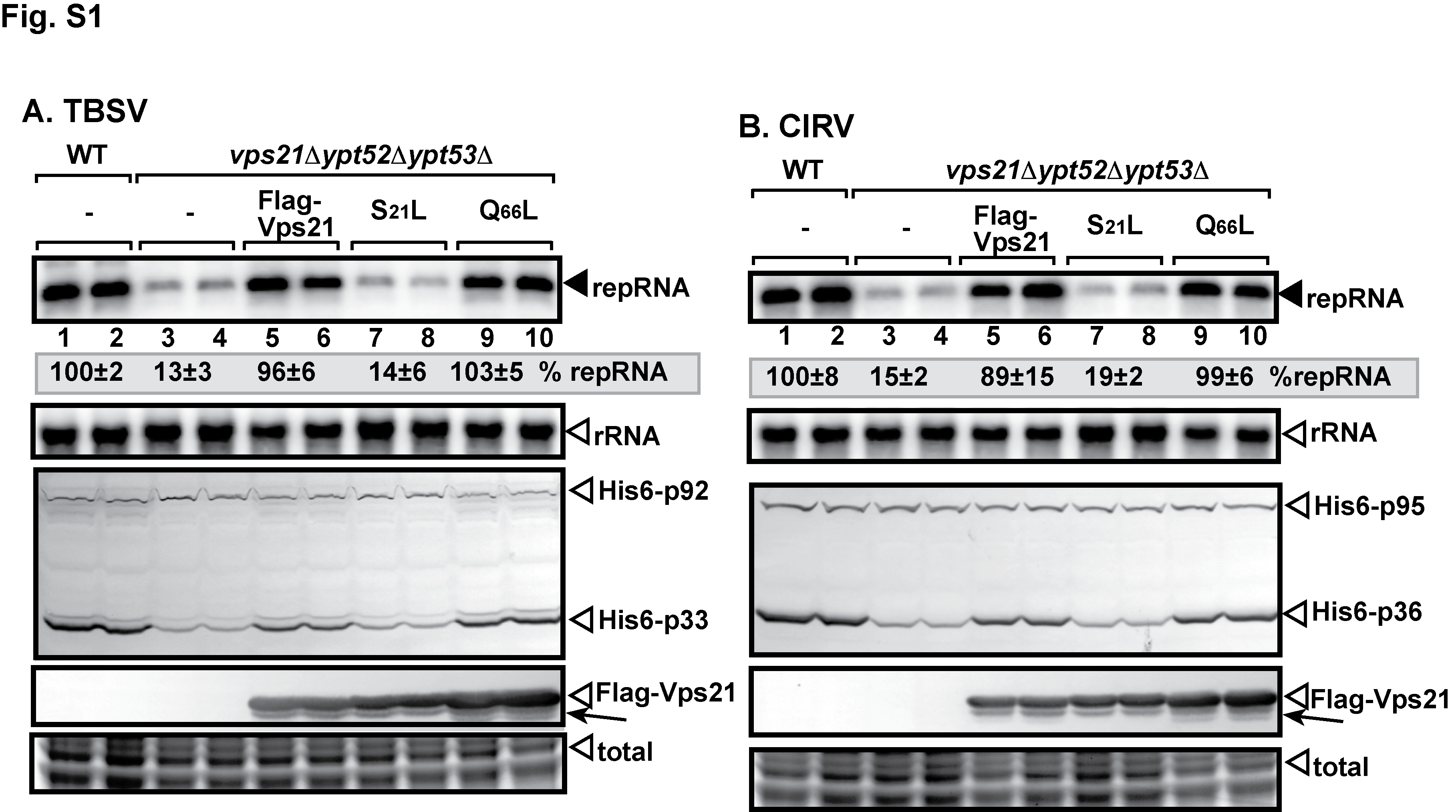

Supplement: S1 Fig — (A) TBSV repRNA accumulation is measured in vps21Δypt52Δypt53Δ yeast expressing His6-p33 and His6-p92 from the galactose-inducible GAL1 promoter, and DI-72(+) repRNA from the galactose-inducible GAL10 promoter. FLAG-tagged Vps21 or its mutants were expressed from the copper-inducible CUP1 promoter based on high copy number plasmids. The yeast cells were pre-cultured for 12 hours at 29°C in 2% glucose SC minimal media, and then for 22 h at 23°C in 2% galactose SC minimal media supplemented with 50 μM CuSO4. Northern blot analysis was used to detect DI-72(+) repRNA accumulation. The accumulation level of DI-72(+) repRNA was normalized based on 18S rRNA levels (second panel from top). Bottom panels: Western blot analysis of the accumulation level of His6-tagged p33, His6-p92 and FLAG-Vps21 proteins using anti-His and anti-FLAG antibodies, respectively. Note that FLAG-Vps21 forms a double band due to prenylation (a lipidation type of posttranslational modification) that is required for binding to the endosomal membrane. The faster migrating band represents the prenylated form of Vps21 (depicted by an arrow), while the unmodified form is depicted by an open arrowhead. The total protein samples were stained with coomassie blue. Each experiment was performed three times. (B) Complementation of CIRV repRNA accumulation in vps21Δypt52Δypt53Δ yeast expressing Vps21p or its mutants. See further details in panel A. (TIF) [file pbio.2000128.s001.tif]

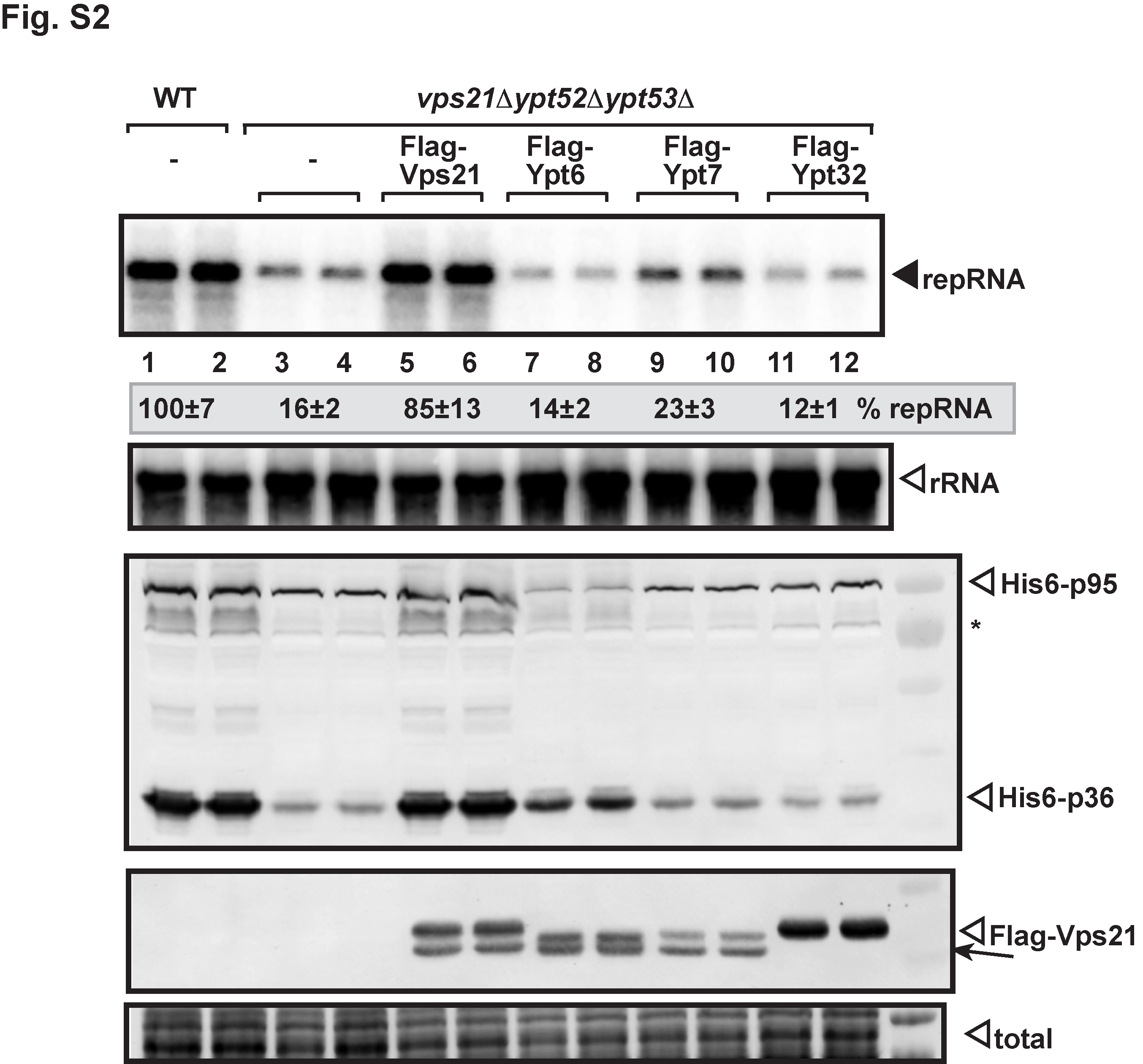

Supplement: S2 Fig — TBSV repRNA accumulation is measured in vps21Δypt52Δypt53Δ yeast expressing His6-p33 and His6-p92 from the galactose-inducible GAL1 promoter, and DI-72(+) repRNA from the galactose-inducible GAL10 promoter. FLAG-tagged Vps21, Ypt6, Ypt7 and Ypt32, respectively, were expressed from the copper-inducible CUP1 promoter based on low copy number plasmids. The yeast cells were pre-cultured for 12 hours at 29°C in 2% glucose SC minimal media, and then for 22 h at 23°C in 2% galactose SC minimal media supplemented with 50 μM CuSO4. Northern blot analysis was used to detect DI-72(+) repRNA accumulation. The accumulation level of DI-72(+) repRNA was normalized based on 18S rRNA levels (second panel from top). Bottom panels: Western blot analysis of the accumulation level of His6-tagged p33, His6-p92 and FLAG-Vps21, Ypt6, Ypt7 and Ypt32 proteins using anti-His and anti-FLAG antibodies, respectively. Note that FLAG-Vps21, Ypt6, and Ypt7 form a double band due to prenylation (a lipidation type of posttranslational modification) that is required for binding to the subcellular membrane. The faster migrating band represents the prenylated forms (depicted by an arrow), while the unmodified form is depicted by an open arrowhead. The total protein samples were stained with coomassie blue. Each experiment was performed three times. (TIF) [file pbio.2000128.s002.tif]

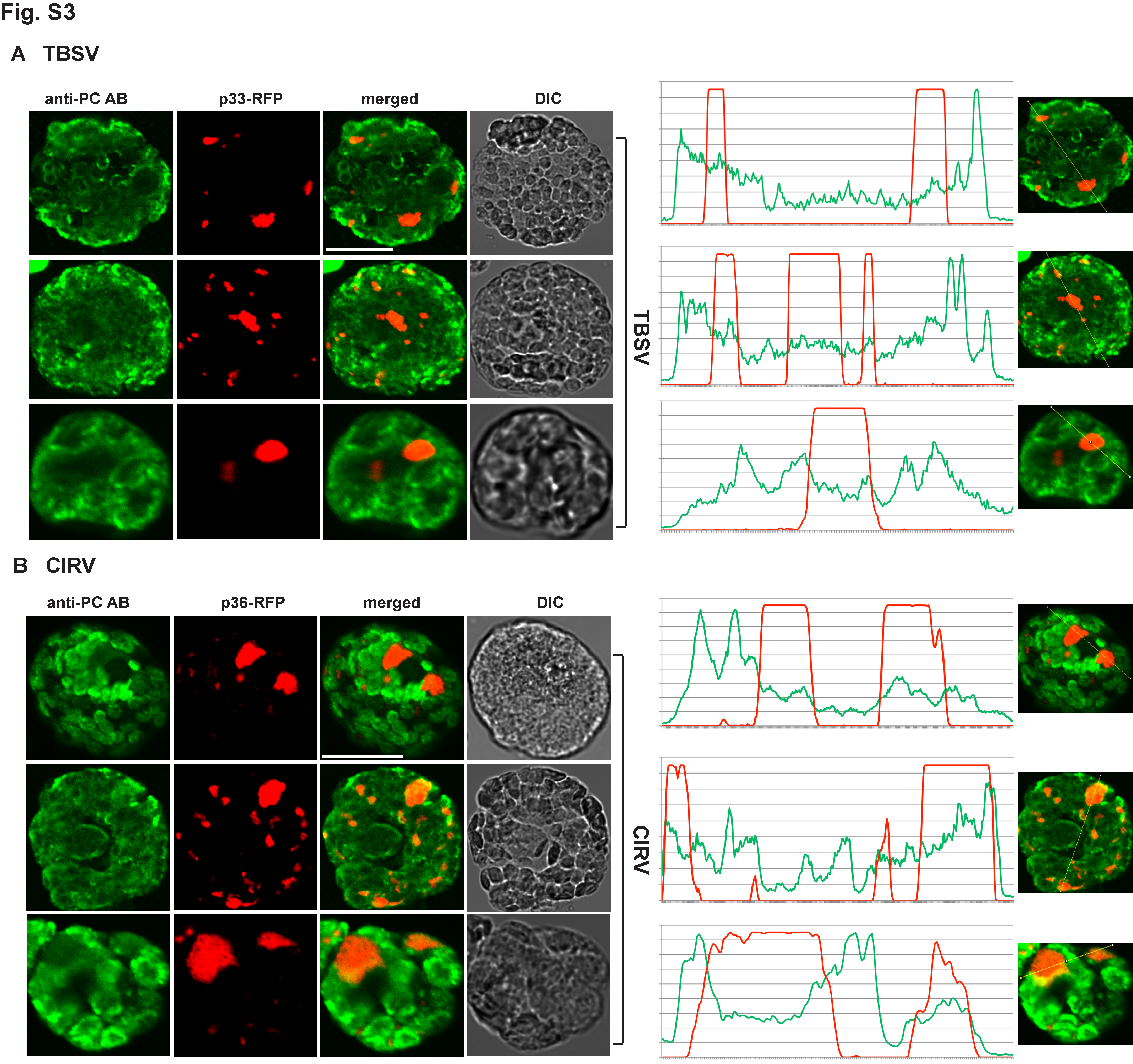

Supplement: S3 Fig — (A-B) The TBSV or CIRV-induced replication compartments are visualized by confocal laser microscopy images. p33-RFP or p36-RFP were expressed based on Agro-infiltration of N. benthamiana leaves. PC distribution was visualized by monoclonal antibody JE-1 and secondary antibody conjugated with Alexa Fluor488. DIC (differential interference contrast) images are shown on the right. Scale bars represent 20 mm. Panels on the right: ImageJ software was used to show the lack of enrichment of PC (green line) in the replication compartment (red line). (TIF) [file pbio.2000128.s003.tif]

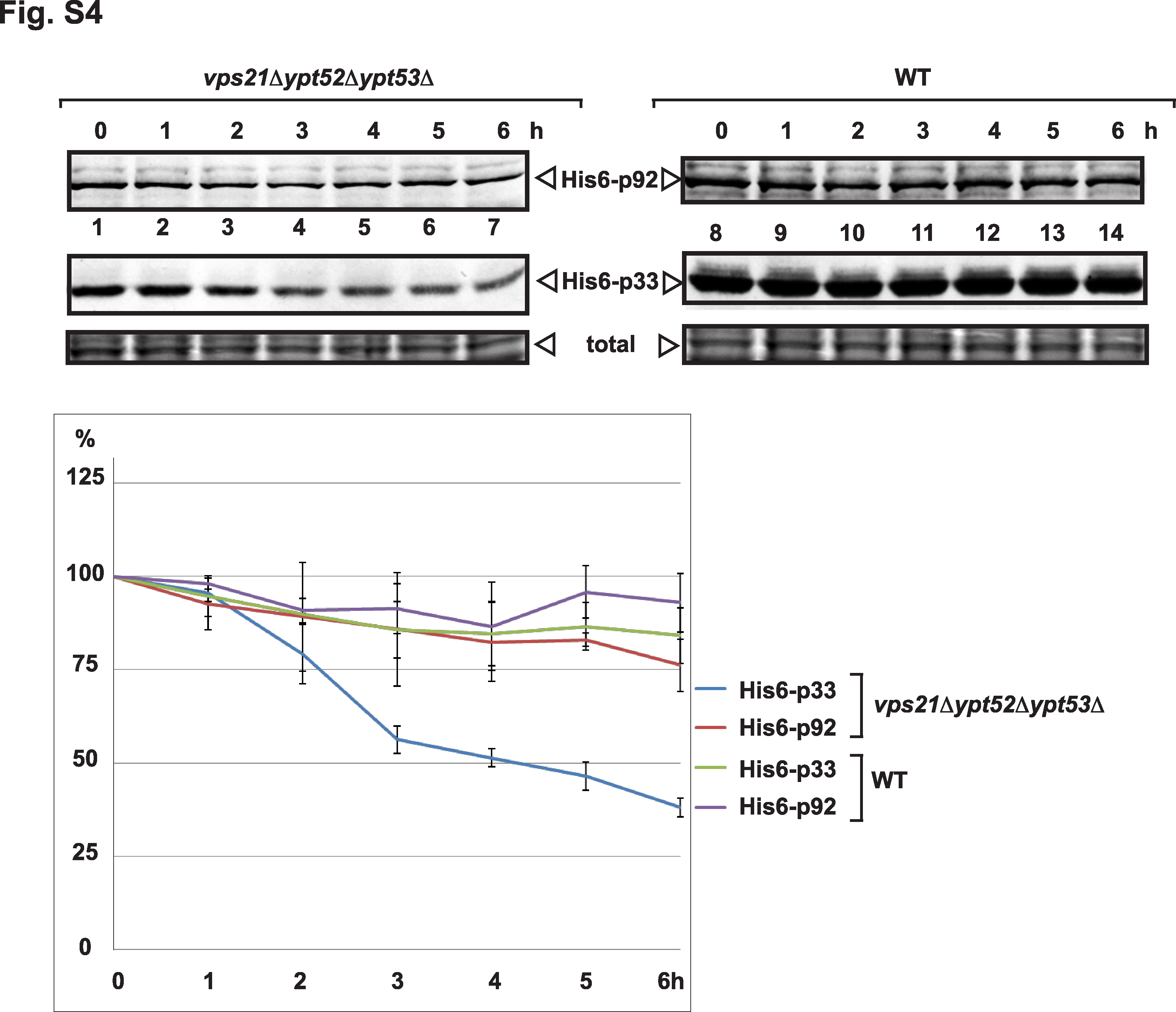

Supplement: S4 Fig — Expression of 6xHis-tagged p33 and 6xHis-p92 in vps21Δypt52Δypt53Δ and wt yeasts was repressed from the GAL1 promoter and via the addition of 100 μg/ml cycloheximide to block new protein synthesis. The total yeast protein samples were analyzed by SDS/PAGE and Western blotting with anti-His antibody to measure the accumulation level of 6xHis-tagged p33 and 6xHis-p92 at the shown time points. (TIF) [file pbio.2000128.s004.tif]

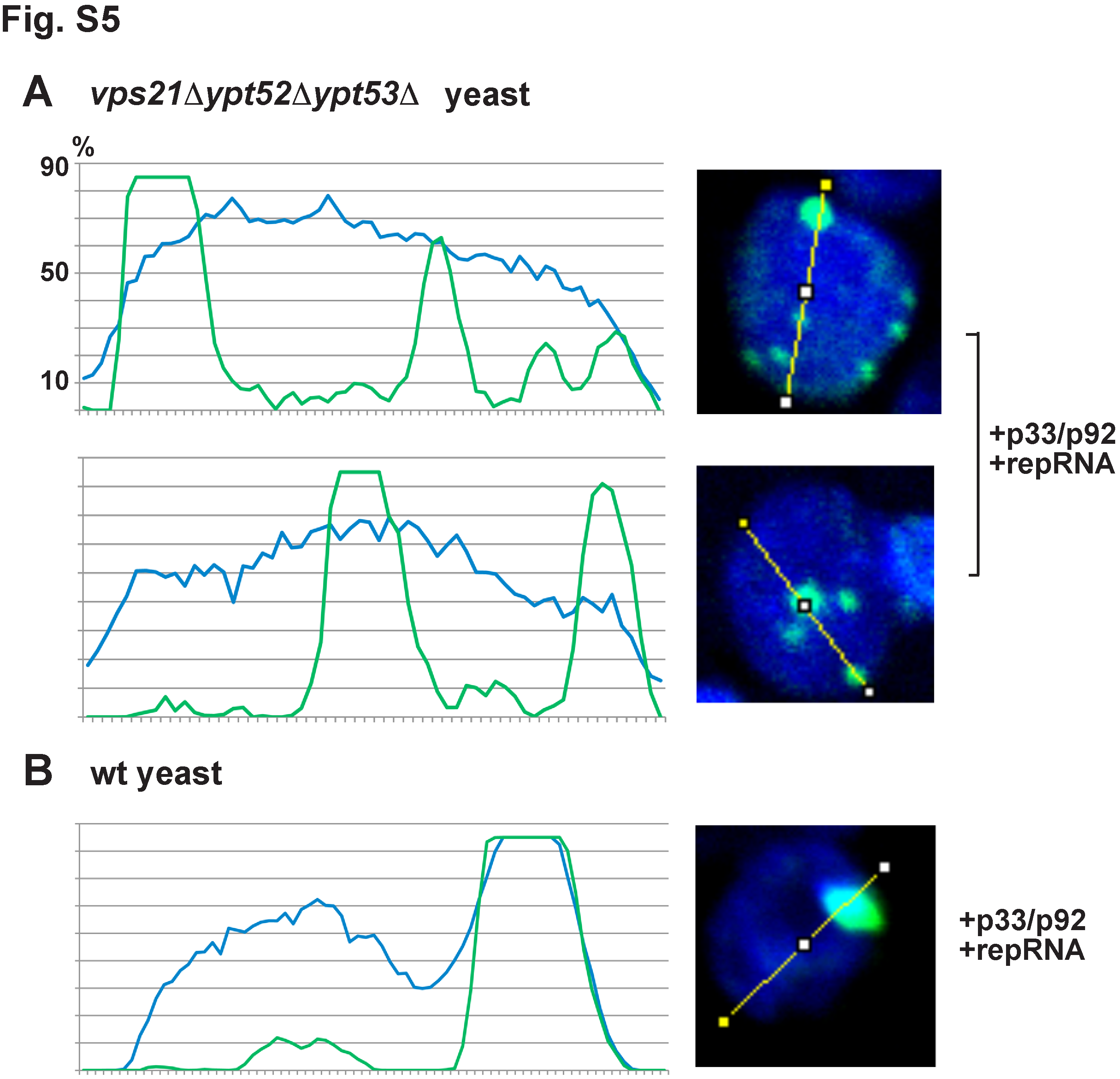

Supplement: S5 Fig — (A) Panels on the left: ImageJ software was used to show the lack of enrichment of PE (blue line) in the replication compartment (green line). Confocal laser microscopy images on the right show PE and TBSV GFP-p33 distribution in vps21Δypt52Δypt53Δ yeast. (B) PE distribution at replication sites in wt yeast. See details in panel A and Fig 3A and 3B. (TIF) [file pbio.2000128.s005.tif]

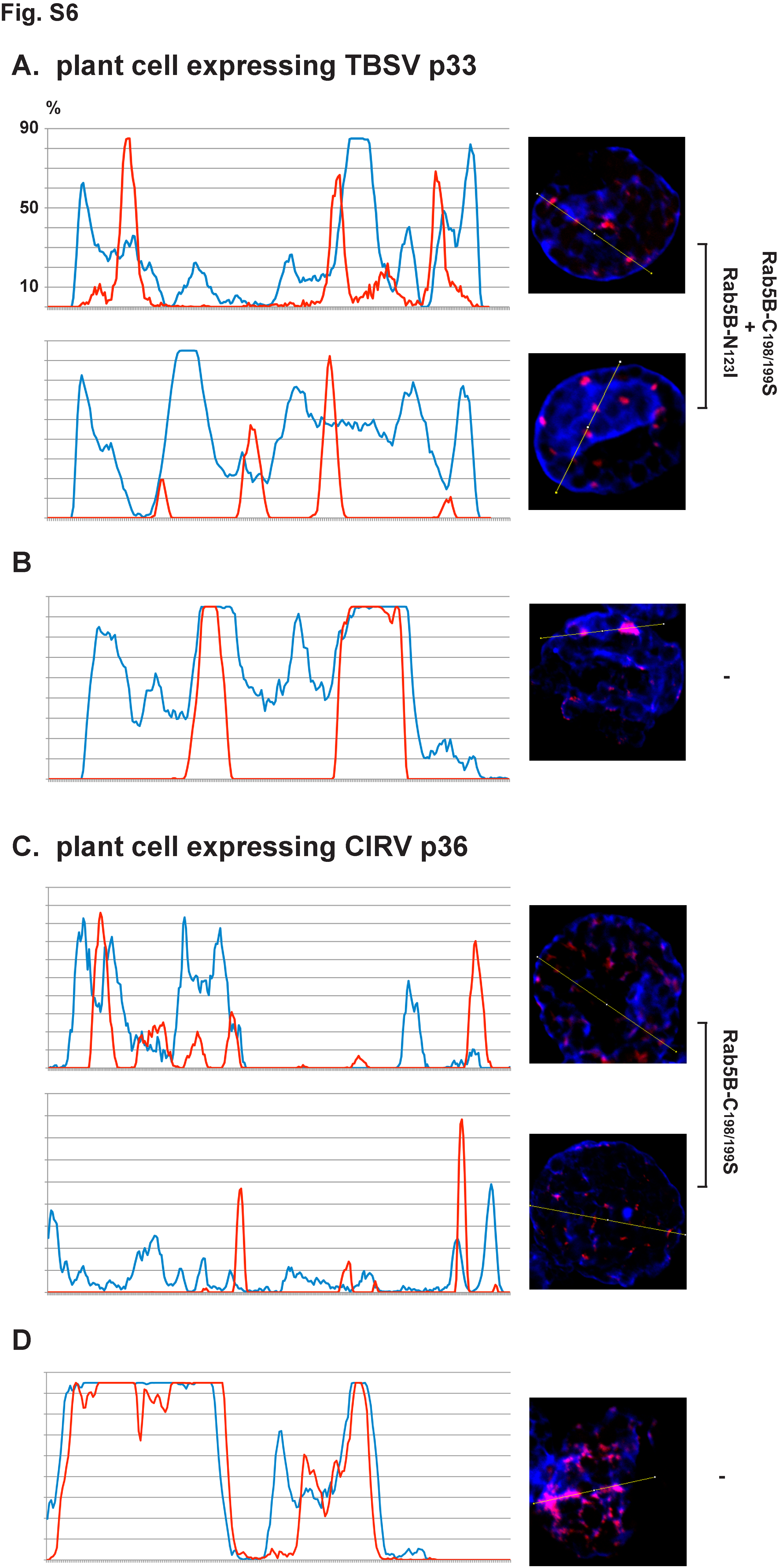

Supplement: S6 Fig — (A-D) Panels on the left: ImageJ software was used to show the enrichment of PE (blue line) in the replication compartment (red line). Confocal laser microscopy images on the right show PE and TBSV p33-RFP and CIRV p36-RFP distribution. See further details in Fig 3D–3H. (TIF) [file pbio.2000128.s006.tif]

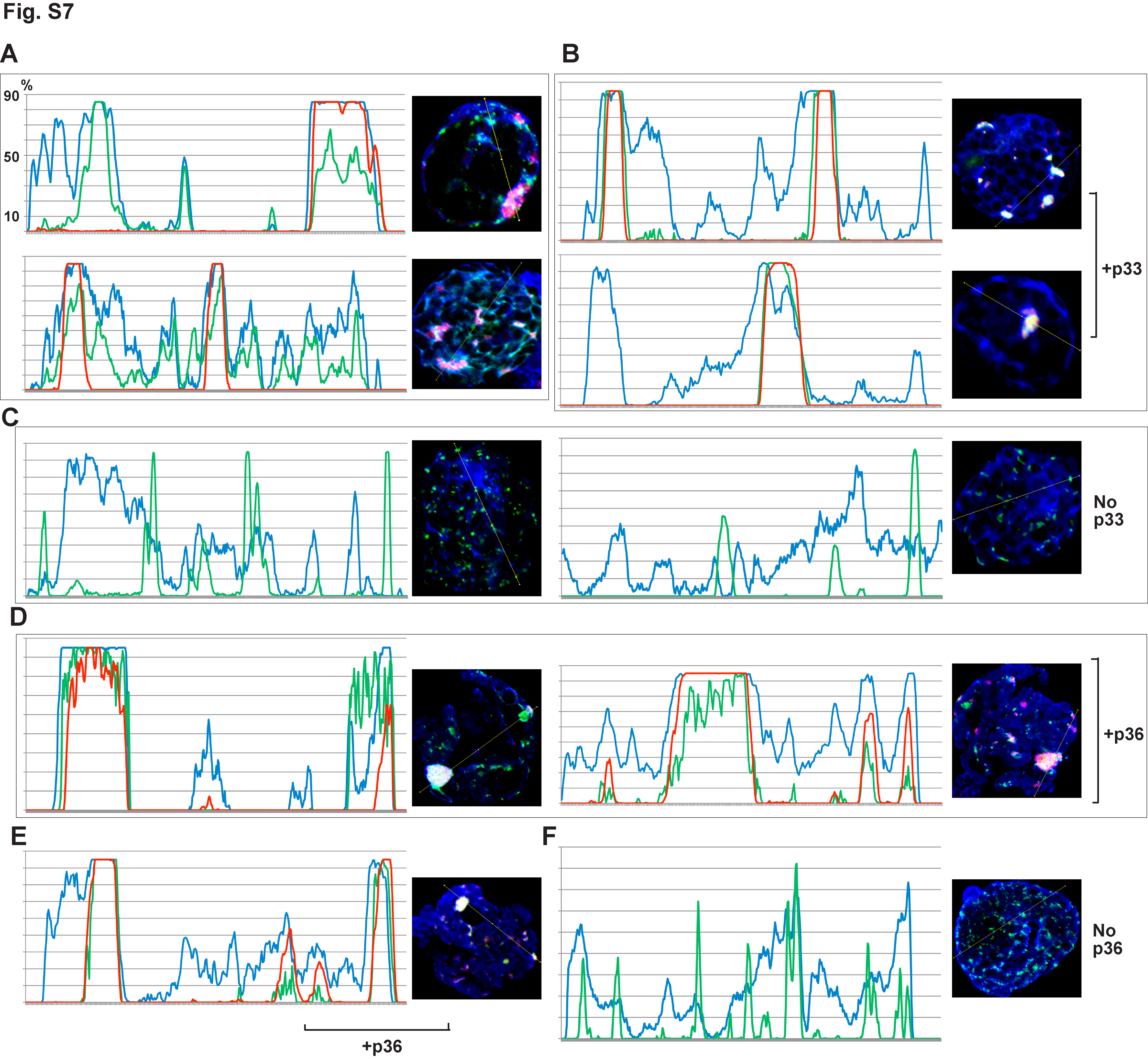

Supplement: S7 Fig — (A-F) Panels on the left: ImageJ software was used to show the enrichment of PE (blue line), AtRab5 (green line) in the replication compartment (red line). Confocal laser microscopy images on the right show GFP-AtRab5, PE detected by duramycin, and TBSV p33-RFP or CIRV p36-RFP distribution. See further details in Fig 4A–4F. (TIF) [file pbio.2000128.s007.tif]

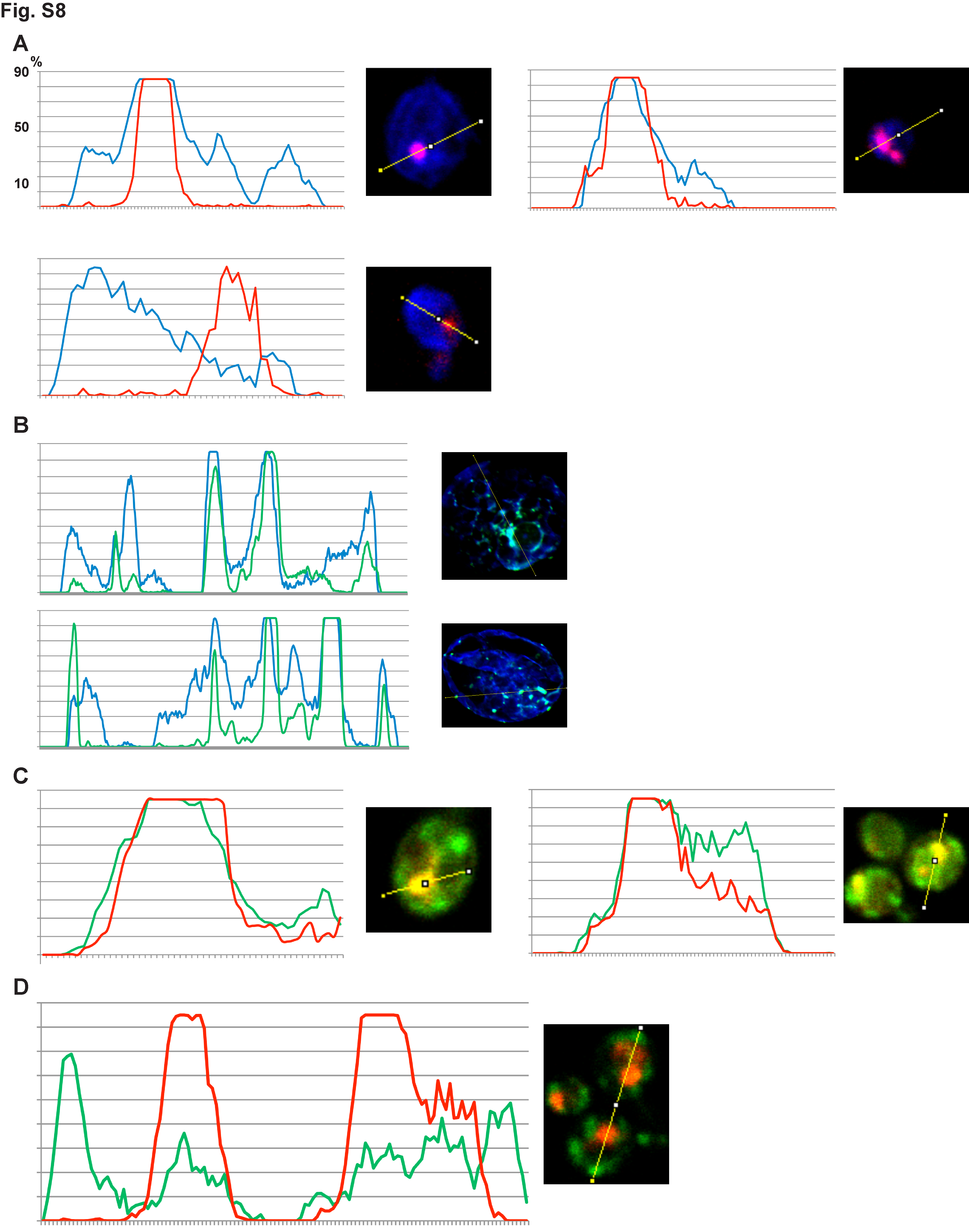

Supplement: S8 Fig — (A) Panels on the left: ImageJ software was used to show the enrichment of PE (blue line) on Vps21 (Rab5)-positive (red line) endosomal membranes in yeast. Confocal laser microscopy images on the right show RFP-Vps21 (or RFP-Tlg1, lower panel), and PE detected by duramycin. See further details in Fig 5A. (B) Panels on the left: ImageJ software was used to show the enrichment of PE (blue line) on AtRab5-positive (green line) endosomal membranes in N. benthamiana cells. Confocal laser microscopy images on the right show GFP-AtRab5 and PE detected by duramycin. See further details in Fig 5B. (C-D) Panels on the left: ImageJ software was used to show the enrichment of exogenous PE (green line) on Vps21 (Rab5)-positive (red line) endosomal membranes, but not in the late Golgi in yeast. Confocal laser microscopy images on the right show RFP-Vps21 (or RFP-Tlg1, panel D) and NBD-PE. (TIF) [file pbio.2000128.s008.tif]

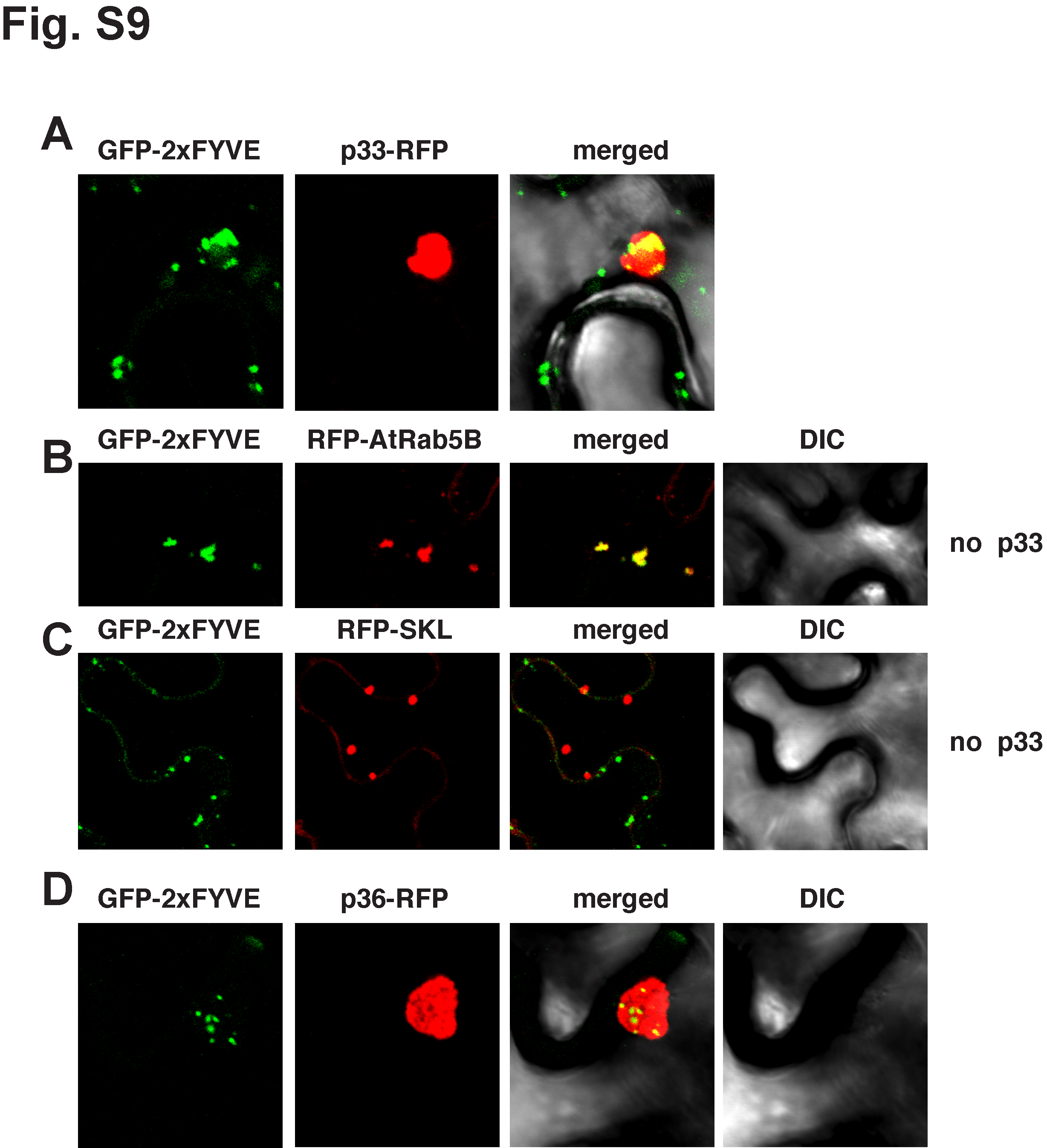

Supplement: S9 Fig — (A) Confocal laser microscopy images show the co-localization of GFP-2xFYVE (PI3P-binding motif) with the TBSV p33-RFP replication protein in subcellular areas in N. benthamiana cells. Note the large replication compartment (representing aggregated peroxisomes) in these cells. (B) Confocal laser microscopy images confirm the localization of PI3P with the Rab5-positive endosomes in the absence of tombusvirus proteins. (C) Confocal laser microscopy images confirm the separate localization of PI3P from GFP-SKL peroxisomal luminal marker protein in the absence of tombusvirus proteins. (D) Confocal laser microscopy images show the co-localization of GFP-2xFYVE (PI3P-binding motif) with the CIRV p36-RFP replication protein in subcellular areas in N. benthamiana cells. Note the large replication compartment (representing aggregated mitochondria) in these cells. (TIF) [file pbio.2000128.s009.tif]

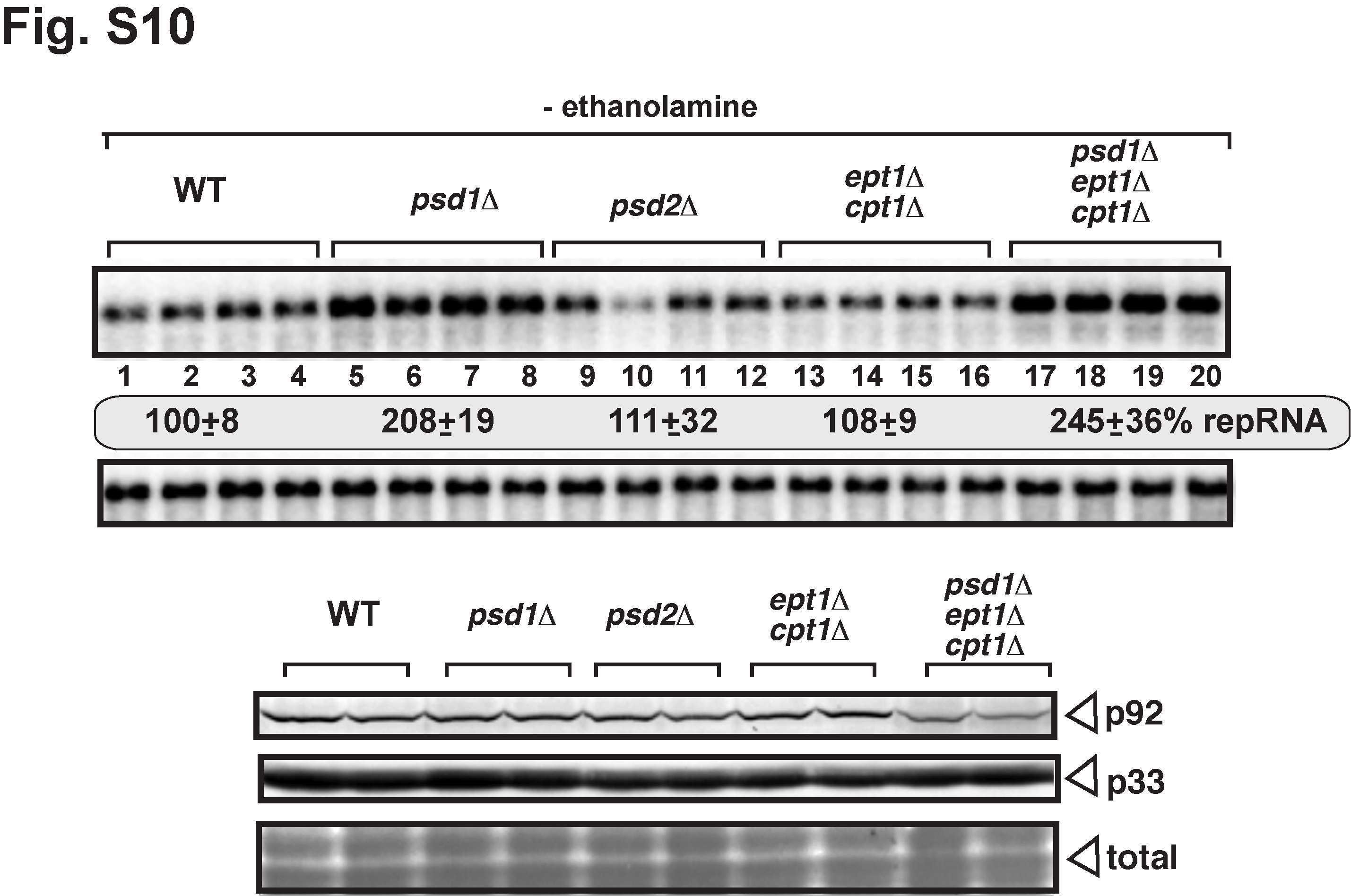

Supplement: S10 Fig — Replication of TBSV in yeast deletion strains grown in media without ethanolamine. Upper Panel: Northern blot of TBSV repRNA and 18S ribosomal RNA. Lower Panel: Western blot of total proteins extracted from different strains tested. TBSV p33 and p92 tagged with His6-tag were detected with an anti-HIS antibody. Total proteins were stained with Ponceau S on PVDF membrane after transfer. Each experiment was repeated. (TIF) [file pbio.2000128.s010.tif]
