# Supplementary material for: Enrichment of Phosphatidylethanolamine in Viral Replication Compartments via Co-opting the Endosomal Rab5 Small GTPase by a Positive-Strand RNA Virus
Source: PLoS Biol. 2016 Oct 19;14(10):e2000128. doi: 10.1371/journal.pbio.2000128 (PMC5070881; doi:10.1371/journal.pbio.2000128)
Supplement: S1 Text — (DOCX) [file pbio.2000128.s011.docx]

**S1 Text.**

**Yeast Strains and expression plasmids.** Parental yeast strain BY4741 (*MATa* *his3Δ1 leu2Δ0 met15Δ0 ura3Δ0*) was purchased from Open Biosystems. Triple gene deletion of *vps21∆ypt52∆ypt53∆* (*MATa his3Δ1 leu2Δ0 met15Δ0 ura3Δ0 vps21Δ::*KanMX4 *ypt52Δ::*hphNT1 *ypt53Δ::*natNT2) was generated from BY4741 parental strain by replacing *VPS21*, *YPT52* and *YPT53* ORF with KanMX4, hphNT1 and natNT2 cassette sequentially using homologous recombination [1]. Yeast strain *vps9∆muk1∆* (*MATa his3Δ1 leu2Δ0 met15Δ0 ura3Δ0* *vps9*Δ::KanMX4 *muk1Δ*::hphNT1) was generated from *vps9∆* single gene deletion strain (YKO collection) by replacing *MUK1* ORF with hphNT1 cassette using homologous recombination [1].

The following high copy number plasmids were prepared: pRS425-pCUP1-Flag-Vps21, pRS425-pCUP1-Flag-Vps21-S21L and pRS425-pCUP1-Flag-Vps21-Q66L. To express Flag-tagged Vps21 under inducible promoter pCUP1, DNA sequence of *CUP1* promoter was PCR-amplified from pEsc-His/Cup-FLAG/ssa1 using primer pair #3039 (CGCGGAGCTCGACATTTGGGCGCTATACGTGCATATGT) and # 5862 (GGAAGATCTCATTTTGCGGCCGCTACAGTTTGTTTTTCTTAATATCTATTTCGA), inserted into SacI/BglII digested pRS315-NFlag, resulting pRS315-pCUP1-NFlag. pRS315-pCUP1-NFlag was digested with SacI/SalI, and the resulting fragment containing pCUP1 promoter and Flag-tag was gel isolated and inserted into SacI/SalI digested pRS425, generating high copy number expression vector pRS425-pCUP1-NFlag. PCR product of yeast *VPS21* (generated using primer pair #5781 and #5782) was digested with BamHI/XhoI, and inserted into BamHI/SalI digested pRS315-pCUP1-NFlag or pRS425-pCUP1-NFlag, resulting pRS315-pCUP1-Flag-Vps21, or pRS425-pCUP1-Flag-Vps21. VPS21 mutations (S21L or Q66L) were introduced into expression vectors using the same approach as for *VPS21* wild type, resulting pRS315-pCUP1-Flag-Vps21-S21L, pRS425-pCUP1-Flag-Vps21-S21L, pRS315-pCUP1-Flag-Vps21-Q66L and pRS425-pCUP1-Flag-Vps21-Q66L.

Yeast *YPT6, YPT7* and *YPT32* sequences were PCR-amplified from yeast total DNA using primer pair #6123 (CGCGGATCCATGAGCAGATCCGGGAAATCATTG) and #6124 (ACGCGTCGACCTAACACTGACAAGCGCTTTGTTCC), #5783 (CGCGGATCCATGTCTTCTAGAAAAAAAAATATTTTGAAAGTAATC) and #5784 (CCGCTCGAGTCAACAGCTACAAGAATTATTTTCTCCATC); and #6125 (CGCGGATCCATGAGCAACGAAGATTACGGATACGACTAC) and #6126 (ACGCGTCGACTTAACAACAGTTGCTGGATTTTTTCTTCTTGTC), digested with BamHI/SalI, BamHI/XhoI or BamHI/SalI individually, and inserted into BamHI/SalI digested pRS315-pCUP1-NFlag, generating pRS315-pCUP1-Flag-Ypt6, pRS315-pCUP1-Flag-Ypt7 and pRS315-pCUP1-Flag-Ypt32.

To generate plasmids for ectopic expression of N-terminal Flag-tagged proteins in plants, sequence of the multi-cloning site and the Flag-tag sequence was PCR-amplified from pRS315-NFlag [2] by primer pair #6070 (CCGTGATCATAAACAATGGCTAGATCTGGCACTAGTGACTACAAGG) / #6071 (ACGCGTCGACGGTATCGATAAGCTTGATATC), followed by digestion of the PCR product with BclI/SalI and then, inserted into BamHI/SalI digested pGD-2x35S-L [3], generating pGD-NFlag. Nucleotide-free mutant (N123I) of Rab5 (*AtRab5B*) was generated by site-directed mutagenesis. Wild type plant Rab5 (*AtRab5B*) and its nucleotide-free mutant (N123I) or prenyl-acceptor mutant (C198,199S) were PCR-amplified by primer pair #6137 (CGCGGATCCATGGCTGCAGCTGGAAACAAGAGC) / #6138 (ACGCGTCGACCTAAGCACAACAAGATGAGCTCACTGCCC) or primer pair #6137 / #6448 (ACGCGTCGACCTAAGCAGAAGAAGATGAGCTCACTGCCC), followed by digestion of the PCR product with BamHI/SalI and then, inserted into BamHI/SalI digested pGD-NFlag, generating pGD-Flag-AtRab5B, pGD-Flag-AtRab5B-N123I or pGD-AtRab5B-C198,199S.

To generate plasmids for expression of N-terminal Red Fluorescent Protein (RFP) or Green Fluorescent Protein (GFP)-tagged proteins, RFP or GFP sequences were PCR-amplified from pGD-CRFP [3] or pSITE-2CA [4] with primer pair #5649 (GGAAGATCTATGGTGTCTAAGGGCGAAGAG) / #5650 (GGACTAGTATTAAGTTTGTGCCCCAGTTTGC) or primer pair #6532 (GGAAGATCTATGGTGAGCAAGGGCGAG) / #6533 (GGACTAGTCTTGTACAGCTCGTCCATGCC), followed by digestion of the PCR product with BglII/SpeI and then, inserted into BglII/SpeI digested pGD-NFlag, generating pGD-NRFP or pGD-NEGFP. To generate plasmid for expression of C-terminal GFP-tagged protein, GFP sequence was PCR-amplified using primer pair #6532 / #6534 (CCGCTCGAGTCACTTGTACAGCTCGTCCATGC), followed by digestion of the PCR product with BglII/XhoI and then, inserted into BglII/XhoI digested pGD-CFlag, resulting pGD-CEGFP.

Wild type plant Rab5 (*AtRab5B*) sequence was PCR-amplified with primer pair #6137 / #6138, and followed by digestion of the PCR product with BamHI/SalI and then, inserted into BamHI/SalI digested pGD-NRFP, generating pGD-RFP-AtRab5B.

To generate plant peroxisomal marker RFP-SKL or GFP-SKL, three amino acids (SKL) were introduced into RFP or rsGFP sequence by PCR-amplification from pGD-CRFP [3] or pYES-rsGFP-T92 [5] using primer pair #5649 / #5653 (CCGCTCGAGTTACAACTTAGAACCGCCACCATTAAGTTTGTGCCCCAGTTTGC) or primer pair #4568 (GGCAGATCTGGTAAAGGAGAAGAACTTTTCACT) / #5654 (CCGCTCGAGTTACAACTTAGAACCGCCACCGAGTCCGGACTTGTATAGTTCA), followed by digestion of the PCR product with BglII/XhoI and then, inserted into BamHI/SalI digested pGD-NFlag, resulting pGD-RFP-SKL or pGD-rsGFP-SKL. Plant peroxisomes were also labelled with C-terminal RFP- or GFP-tagged peroxisomal membrane protein Pex13. *AtPex13* sequence was PCR-amplified from *Arabidopsis* cDNA with primer pair #6175 (CGCGGATCCTAAACAATGGCGTCTCAGCCTGCAGG) / #6176 (GGCATGCATGTTGCCCCATACATTGTCCCAACC), followed by digestion of the PCR product with BamHI/NsiI and inserted into BamHI/PstI digested pGD-CRFP or pGD-CEGFP, generating pGD-AtPex13-RFP or pGD-AtPex13-EGFP.

To generate mitochondrion marker mito-RFP/GFP [6] or AtTim21-RFP/GFP [7], the known mitochondria import sequence from yeast *COX4* gene [6] was PCR-amplified from yeast total DNA using primer pair #6536 (CGCGGATCCTAAACAATGCTTTCACTACGTCAATCTATAAG) / #6537 (GCCCTGCAGGGGTTTTTGCTGAAGCAGATATC), followed by digestion of the PCR product with BamHI/PstI and then, inserted into BamHI/PstI digested pGD-CRFP or pGD-CEGFP, resulting pGD-Cox4-RFP (mito-RFP) or pGD-Cox4-EGFP (mito-GFP). Plant translocase of the Inner Mitochondrial membrane 21 (*AtTim21*, At4g00026) was PCR-amplified from *Arabidopsis* total cDNA using primer pair #6181 (CCGTGATCATAAACAATGATGATGATGAATCTTTTAAGGAGATCAGC) / #6182 (GCCCTGCAGGGCAGGCAAATAAGACTCCAGCATC), followed by digestion of the PCR product with BclI/PstI and then, inserted into BamHI/PstI digested pGD-CRFP or pGD-CEGFP, resulting pGD-AtTim21-RFP or pGD-AtTim21-EGFP.

For Bimolecular fluorescence complementation (BiFC) assay, N-terminal half of Yellow Fluorescent Protein (YFP) was PCR-amplified from pSITE-3NB [4] using primer pair #5905 (GGAAGATCTATGGTGAGCAAGGGCGAG) / #6069 (GCGCGGATCCGTCCTCGATGTTGTGGC), followed by digestion of the PCR product with BamHI, and then ligated with MBP fragments digested BamHI, which is PCR-amplified from pMAL-c2X (NEB) using primer pair #6188 (CGCGGATCCTAAACAATGGCTAAAATCGAAGAAGGTAAACTG) / #6189 (GCGTCGACTCATGAAATCCTTCCCTCGATC). The nYFP-MBP fusion protein was PCR-amplified using primer pair #5905 / #6189, followed by digestion of the PCR product with BglII/SalI and then, inserted into BamHI/SalI digested pGD-2x35S-L [3], resulting pGD-nYFP-MBP. The PCR products of *AtRab5B* digested with BamHI/SalI were inserted into pGD-nYFP-MBP digested with BamHI/SalI, resulting in pGD-nYFP-AtRab5B. For C-terminal half of YFP tagged TBSV p33 or CIRV p36, C-terminal half of YFP was PCR-amplified with primer pair #5908 (GGAAGATCTATGGGCAGCGTGCAGCTC) / #5910 (CCGCTCGAGTCACTTGTACAGCTCGTCCATGCC) from pSITE-3NB [4], followed by digestion of the PCR product with BglII/XhoI and then, inserted into pGD-CFlag digested with BglII/XhoI, resulting in pGD-C-cYFP. The DNA sequences of TBSV p33 or CIRV p36 were PCR-amplified from pESC-T33/DI72 [5] or pESC-C36/DI1 [2] using primer pair #6075 (CGCGGATCCTAAACAATGGAGACCATCAAGAGAATG) /#5837 (GCCCTGCAGTTTGACACCCAGGGACTCCTG) or #6184 (CGCGGATCCTAAACAATGGAGGGTTTGAAGGCTG) /#6185 (GGCATGCATTTTGACACCGAGGGATTCC), followed by digestion of the PCR product with BamHI/PstI and BamHI/NsiI, respectively, and then, separately inserted into pGD-C-cYFP digested with BamHI/PstI, resulting in pGD-T33-cYFP or pGD-C36-cYFP.

To express Blue Fluorescent Protein (BFP)-tagged TBSV p33 or CIRV p36 for protein expression in plant leaves, BFP sequence was PCR-amplified from Gateway TagBFP-AS-C entry clone (Evrogen) using primer pair #5673 (GGAAGATCTATGTCTGAATTGATTAAAGAGAATATGC) / #5675 (CCGCTCGAGTTAGTTCAATTTGTGTCCTAACTTAGAAGG), followed by digestion of the PCR product with BglII/XhoI and inserted into pGD-CFlag [3], resulting in pGD-CBFP. BamHI/PstI digested TBSV p33 sequence or BamHI/NsiI digested CIRV p36 sequence were inserted into BamHI/PstI digested pGD-CBFP, resulting in pGD-T33-BFP or pGD-C36-BFP. To express BFP-tagged TBSV p33 or CIRV p36, BFP sequence was PCR-amplified from Gateway TagBFP-AS-C entry clone (Evrogen) using primer pair #5673 / #5694 (CGCGGATCCGTTCAATTTGTGTCCTAACTTAGAAG), followed by digestion of the PCR product with BamHI and then, ligated with BamHI digested TBSV p33 or CIRV p36 PCR products. The DNA sequences encoding BFP-p33 or BFP-p36 were PCR-amplified using primer pair #5673 / #810 (GGAGCTCGAGCTATTTGACACCCAGGGAC) or primer pair #5673 / #3230 (CCGCTCGAGCTATTTGACACCGAGGGATT), followed by digestion of the PCR products with BamHI/XhoI and then, inserted into BamHI/XhoI digested pESC-T33/DI72 [2], resulting in pESC-BFP-T33/DI72 or pESC-BFP-C36/DI72.

For expression of RFP-Vps21 in yeast, BamHI/PstI digested PCR product of RFP (amplified by primer pair #5649/#5650 from pGD-NRFP) was inserted into pRS315-NFlag [2], resulting in pRS315-NRFP. *VPS21* sequence was PCR-amplified from yeast total DNA using primer pair #5781 (CGCGGATCCATGAACACATCAGTCACTTCCATAAAGTTG) / #5782 (CCGCTCGAGCTAACAACTGCAAGCACTGTTTGC), followed by digestion of the PCR product with BamHI/XhoI and then, inserted into BamHI/SalI digested pRS315-NRFP, resulting in pRS315-RFP-Vps21. To express Flag-tagged Vps21 under inducible promoter *pCUP1*, the DNA sequence of *CUP1* promoter was PCR-amplified from pEsc-His/Cup-FLAG/ssa1 [8] using primer pair #3039 (CGCGGAGCTCGACATTTGGGCGCTATACGTGCATATGT) / # 5862 (GGAAGATCTCATTTTGCGGCCGCTACAGTTTGTTTTTCTTAATATCTATTTCGA), and then, inserted into SacI/BglII digested pRS315-NFlag, resulting in pRS315-pCUP1-NFlag. The PCR product of yeast *VPS21* (obtained by using primer pair #5781 / #5782) was digested with BamHI/XhoI, and then, inserted into BamHI/SalI digested pRS315-pCUP1-NFlag, resulting in pRS315-pCUP1-Flag-Vps21. VPS21 mutations (S21L or Q66L) were introduced into Vps21 expression vector, generating pRS315-pCUP1-Flag-Vps21-S21L or pRS315-pCUP1-Flag-Vps21-Q66L.

**Preparation of CFE, membrane fraction (P40) and soluble fraction (S40).** The yeast extracts (CFE) from yeast strains BY4741 and *vps21∆ypt52∆ypt53∆* (Rab5 deletion strain) were prepared according to [9]. CFE was centrifuged at 40,000 g/ 100,000 g for 1 h to isolate the soluble fraction (S40 or S100) from total membrane fraction (P40 or P100). Collected fractions were stored at -80 °C immediately after isolation.

**MBP pull-down assay.** DNA fragment of yeast *VPS21* was tagged with 10xHis-tag via PCR amplification from total yeast DNA using primer pair #6357 (GGAATTCCATATGAACACATCAGTCACTTCC)/ #6358 (CCGCTCGAGTTAATGATGATGATGATGATGATGATGATGATGACCACTGCTAGCACTGTT), followed by digestion of the PCR product with NdeI/XhoI and then, inserted into NdeI/XhoI digested pET30a (Novagen), resulting in pET30a-Vps21-10xHis. NdeI site within the AtRab5B cDNA was removed by site directed mutagenesis on pGD-Flag-AtRab5B using primer pair #6433 (CAGAGGATGCACAAACATACGCTCAGGAAAACGGTC)/ #6434 (GACCGTTTTCCTGAGCGTATGTTTGTGCATCCTCTG). These changes did not affect the amino acids sequence in AtRab5B. The obtained cDNA of AtRab5B (without NdeI site) was PCR-amplified using primer pair #6435 (GGAATTCCATATGGCTGCAGCTGGAAAC)/ #6436 (CCGCTCGAGTTAATGATGATGATGATGATGATGATGATGATGAGCACCAGAAGATGAGCT), followed by digestion of the PCR product with NdeI/XhoI, and then, inserted into NdeI/XhoI digested pET30a (Novagen), resulting in pET30a-AtRab5B-10xHIS. Since the two C-terminal cysteines in Vps21 (C208S, C210G) and AtRab5B (C198S, C199G) are prenyl-acceptor for membrane association, they were mutated to preclude possible disulfide bond formation during pull-down assay.

pET30a-Vps21-10xHIS and pET30a-AtRab5B-10xHIS were transformed separately into *E. Coli* Strain BL21 (DE3) CodonPlus. Recombinant protein expression was induced by 0.3 mM isopropyl β-D-thiogalactopyranoside (IPTG) at 16 °C for 16 hours, and the cells were collected by centrifugation at 5,000 rpm at 4 °C for 15 min to remove medium before storage at -80 °C. *E. Coli* cells were lysed by sonication on ice in 20 mM HEPES-KOH, pH 7.4, 500 mM NaCl, 3 mM MgCl_2_, 2 mM β-mercaptoethanol and 1 mM GTP. Soluble proteins were collected via centrifugation at 20,000 xg for 30 min, and then used for MBP pull-down assay. pMAL-c2X, pMAL-33C or pMAL-36C encoding MBP, MBP-p33C and MBP-p36C respectively was transformed individually into *E. Coli* Strain BL21 (DE3) CodonPlus. Protein induction procedure and pull-down assay were described previously [2].

**Analysis of protein-protein interaction by split-ubiquitin based yeast two hybrid assay.** DNA fragment of yeast *VPS21* gene or its mutants was PCR-amplified using primer pair #5781 (CGCGGATCCATGAACACATCAGTCACTTCCATAAAGTTG)/ #5782 (CCGCTCGAGCTAACAACTGCAAGCACTGTTTGC), followed by digestion of the PCR product with BamHI/Xho, and inserted into BamHI/SalI digested pPR3N-RE [10], generating pPR3N-Vps21, pPR3N-Vps21-S21L and pPR3N-Vps21-Q66L. The bait constructs, pGAD-BT2-N-His36 or pGAD-BT2-N-His33 [10,11], were transformed with one of the prey constructs, pPR3N-RE, pPR3N-Vps21, pPR3N-Vps21-S21L or pPR3N-Vps21-Q66L, into yeast strain NMY51. Cells were plated onto Trp^-^/Leu^-^ (TL^-^) synthetic minimal medium plates for plasmid selection. Yeast colonies were re-suspended in water and spotted onto Trp^-^/Leu^-^/His^-^/Ade^-^ (TLHA^-^) plates for 2 days to detect bait-prey interactions.

**p33 and p92 stability assay in yeast**. 6xHis-tagged p33 and p92 were expressed under *GAL1* promoter from the plasmids pESC-T33/DI72 and pYES-T92. Wild type or mutant yeast cells were cultured overnight within synthetic complete medium lacking urea and histidine supplemented with 2% glucose, and then cells were grown in synthetic complete medium lacking urea and histidine supplemented with 2% galactose for 22 hours at 29 °C to induce p33 and p92 expression. Then, cells were washed and incubated at 23 °C with synthetic complete medium lacking urea and histidine supplemented with 2% glucose to inhibit mRNA transcription from *GAL1* promoter and with 100 μg/ml cycloheximide to block new protein synthesis [12]. Equal amounts of yeast cells were collected at 0, 1, 2, 3, 4, 5 and 6 hours time points. The total yeast protein samples were analyzed by SDS/PAGE and Western blotting with anti-His antibody to measure the accumulation level of 6xHis-tagged p33 and p92.

1. Janke C, Magiera MM, Rathfelder N, Taxis C, Reber S, et al. (2004) A versatile toolbox for PCR-based tagging of yeast genes: new fluorescent proteins, more markers and promoter substitution cassettes. Yeast 21: 947-962.

2. Xu K, Lin JY, Nagy PD (2014) The hop-like stress-induced protein 1 cochaperone is a novel cell-intrinsic restriction factor for mitochondrial tombusvirus replication. J Virol 88: 9361-9378.

3. Nawaz-Ul-Rehman MS, Prasanth KR, Xu K, Sasvari Z, Kovalev N, et al. (2016) Viral Replication Protein Inhibits Cellular Cofilin Actin Depolymerization Factor to Regulate the Actin Network and Promote Viral Replicase Assembly. PLoS Pathog 12: e1005440.

4. Chakrabarty R, Banerjee R, Chung SM, Farman M, Citovsky V, et al. (2007) PSITE vectors for stable integration or transient expression of autofluorescent protein fusions in plants: probing Nicotiana benthamiana-virus interactions. Mol Plant Microbe Interact 20: 740-750.

5. Xu K, Huang TS, Nagy PD (2012) Authentic in vitro replication of two tombusviruses in isolated mitochondrial and endoplasmic reticulum membranes. J Virol 86: 12779-12794.

6. Kohler RH, Zipfel WR, Webb WW, Hanson MR (1997) The green fluorescent protein as a marker to visualize plant mitochondria in vivo. Plant J 11: 613-621.

7. Murcha MW, Kubiszewski-Jakubiak S, Wang Y, Whelan J (2014) Evidence for interactions between the mitochondrial import apparatus and respiratory chain complexes via Tim21-like proteins in Arabidopsis. Front Plant Sci 5: 82.

8. Pogany J, Stork J, Li Z, Nagy PD (2008) In vitro assembly of the Tomato bushy stunt virus replicase requires the host Heat shock protein 70. Proc Natl Acad Sci U S A 105: 19956-19961.

9. Pogany J, Nagy PD (2008) Authentic replication and recombination of Tomato bushy stunt virus RNA in a cell-free extract from yeast. J Virol 82: 5967-5980.

10. Mendu V, Chiu M, Barajas D, Li Z, Nagy PD (2010) Cpr1 cyclophilin and Ess1 parvulin prolyl isomerases interact with the tombusvirus replication protein and inhibit viral replication in yeast model host. Virology 406: 342-351.

11. Li Z, Barajas D, Panavas T, Herbst DA, Nagy PD (2008) Cdc34p ubiquitin-conjugating enzyme is a component of the tombusvirus replicase complex and ubiquitinates p33 replication protein. J Virol 82: 6911-6926.

12. Barajas D, Martin IF, Pogany J, Risco C, Nagy PD (2014) Noncanonical Role for the Host Vps4 AAA+ ATPase ESCRT Protein in the Formation of Tomato Bushy Stunt Virus Replicase. PLoS Pathog 10: e1004087.

**S1 Fig**

**Complementation of tombusvirus replication by Vps21 in yeast lacking the three Rab5 orthologous genes. (A)** TBSV repRNA accumulation is measured in *vps21Δypt52Δypt53Δ* yeast expressing His_6_-p33 and His_6_-p92 from the galactose-inducible *GAL1* promoter, and DI-72(+) repRNA from the galactose-inducible *GAL10* promoter. FLAG-tagged Vps21 or its mutants were expressed from the copper-inducible *CUP1* promoter based on high copy number plasmids. The yeast cells were pre-cultured for 12 hours at 29 ºC in 2% glucose SC minimal media, and then for 22 h at 23 ºC in 2% galactose SC minimal media supplemented with 50 µM CuSO_4_. Northern blot analysis was used to detect DI-72(+) repRNA accumulation. The accumulation level of DI-72(+) repRNA was normalized based on 18S rRNA levels (second panel from top). Bottom panels: Western blot analysis of the accumulation level of His_6_-tagged p33, His_6_-p92 and FLAG-Vps21 proteins using anti-His and anti-FLAG antibodies, respectively. Note that FLAG-Vps21 forms a double band due to prenylation (a lipidation type of posttranslational modification) that is required for binding to the endosomal membrane. The faster migrating band represents the prenylated form of Vps21 (depicted by an arrow), while the unmodified form is depicted by an open arrowhead. The total protein samples were stained with coomassie blue. Each experiment was performed three times. **(B)** Complementation of CIRV repRNA accumulation in *vps21Δypt52Δypt53Δ* yeast expressing Vps21p or its mutants. See further details in panel A.

**S2 Fig**

**Lack of complementation of tombusvirus replication by various yeast Rab GTPases in yeast lacking the three Rab5 orthologous genes.** TBSV repRNA accumulation is measured in *vps21Δypt52Δypt53Δ* yeast expressing His_6_-p33 and His_6_-p92 from the galactose-inducible *GAL1* promoter, and DI-72(+) repRNA from the galactose-inducible *GAL10* promoter. FLAG-tagged Vps21, Ypt6, Ypt7 and Ypt32, respectively, were expressed from the copper-inducible *CUP1* promoter based on low copy number plasmids. The yeast cells were pre-cultured for 12 hours at 29 ºC in 2% glucose SC minimal media, and then for 22 h at 23 ºC in 2% galactose SC minimal media supplemented with 50 µM CuSO_4_. Northern blot analysis was used to detect DI-72(+) repRNA accumulation. The accumulation level of DI-72(+) repRNA was normalized based on 18S rRNA levels (second panel from top). Bottom panels: Western blot analysis of the accumulation level of His_6_-tagged p33, His_6_-p92 and FLAG-Vps21, Ypt6, Ypt7 and Ypt32 proteins using anti-His and anti-FLAG antibodies, respectively. Note that FLAG-Vps21, Ypt6, and Ypt7 form a double band due to prenylation (a lipidation type of posttranslational modification) that is required for binding to the subcellular membrane. The faster migrating band represents the prenylated forms (depicted by an arrow), while the unmodified form is depicted by an open arrowhead. The total protein samples were stained with coomassie blue. Each experiment was performed three times.

**S3 Fig**

**Lack of PC enrichment within tombusvirus replication compartment in plant cells. (A-B)** The TBSV or CIRV-induced replication compartments are visualized by confocal laser microscopy images. p33-RFP or p36-RFP were expressed based on Agro-infiltration of *N. benthamiana* leaves. PC distribution was visualized by monoclonal antibody JE-1 and secondary antibody conjugated with Alexa Fluor488. DIC (differential interference contrast) images are shown on the right. Scale bars represent 20 μm. Panels on the right: ImageJ software was used to show the lack of enrichment of PC (green line) in the replication compartment (red line).

**S4 Fig**

**Decreased stability of TBSV replication proteins in yeast lacking the three Rab5 orthologous genes.** Expression of 6xHis-tagged p33 and 6xHis-p92 in *vps21Δypt52Δypt53Δ* and wt yeasts was repressed from the *GAL1* promoter and via the addition of 100 μg/ml cycloheximide to block new protein synthesis. The total yeast protein samples were analyzed by SDS/PAGE and Western blotting with anti-His antibody to measure the accumulation level of 6xHis-tagged p33 and 6xHis-p92 at the shown time points.

**S5 Fig**

**Lack of enrichment of PE at TBSV replication sites in *vps21Δypt52Δypt53Δ* yeast.** (A) Panels on the left: ImageJ software was used to show the lack of enrichment of PE (blue line) in the replication compartment (green line). Confocal laser microscopy images on the right show PE and TBSV GFP-p33 distribution in *vps21Δypt52Δypt53Δ* yeast. (B) PE distribution at replication sites in wt yeast. See details in panel A and Fig. 3A-B.

**S6 Fig**

**Measuring PE enrichment at TBSV replication sites in *N. benthamiana* cells expressing dominant negative mutants of AtRab5 proteins.** (A-D) Panels on the left: ImageJ software was used to show the enrichment of PE (blue line) in the replication compartment (red line). Confocal laser microscopy images on the right show PE and TBSV p33-RFP and CIRV p36-RFP distribution. See further details in Figure 3D-H.

**S7 Fig**

**Measuring Rab5 colocalization with PE enriched TBSV replication compartment in *N. benthamiana* cells.** (A-F) Panels on the left: ImageJ software was used to show the enrichment of PE (blue line), AtRab5 (green line) in the replication compartment (red line). Confocal laser microscopy images on the right show GFP-AtRab5, PE detected by duramycin, and TBSV p33-RFP or CIRV p36-RFP distribution. See further details in Figure 4A-F.

**S8 Fig**

**Measuring PE-richness of Rab5-positive endosomes in the absence of tombusviruses in yeast and *N. benthamiana* cells.** (A) Panels on the left: ImageJ software was used to show the enrichment of PE (blue line) on Vps21 (Rab5)-positive (red line) endosomal membranes in yeast. Confocal laser microscopy images on the right show RFP-Vps21 (or RFP-Tlg1, lower panel), and PE detected by duramycin. See further details in Figure 5A. (B) Panels on the left: ImageJ software was used to show the enrichment of PE (blue line) on AtRab5-positive (green line) endosomal membranes in *N. benthamiana* cells. Confocal laser microscopy images on the right show GFP-AtRab5 and PE detected by duramycin. See further details in Figure 5B. (C-D) Panels on the left: ImageJ software was used to show the enrichment of exogenous PE (green line) on Vps21 (Rab5)-positive (red line) endosomal membranes, but not in the late Golgi in yeast. Confocal laser microscopy images on the right show RFP-Vps21 (or RFP-Tlg1, panel D) and NBD-PE.

**S9 Fig**

**PI3P is partly co-localized with the tombusvirus replication compartment in plant cells.** (A) Confocal laser microscopy images show the co-localization of GFP-2xFYVE (PI3P-binding motif) with the TBSV p33-RFP replication protein in subcellular areas in *N. benthamiana* cells. Note the large replication compartment (representing aggregated peroxisomes) in these cells. (B) Confocal laser microscopy images confirm the localization of PI3P with the Rab5-positive endosomes in the absence of tombusvirus proteins. (C) Confocal laser microscopy images confirm the separate localization of PI3P from GFP-SKL peroxisomal luminal marker protein in the absence of tombusvirus proteins. (D) Confocal laser microscopy images show the co-localization of GFP-2xFYVE (PI3P-binding motif) with the CIRV p36-RFP replication protein in subcellular areas in *N. benthamiana* cells. Note the large replication compartment (representing aggregated mitochondria) in these cells.

**S10 Fig**

**Altered TBSV replication in PE synthesis pathway deletion yeast strains.** Replication of TBSV in yeast deletion strains grown in media without ethanolamine. Upper Panel: Northern blot of TBSV repRNA and 18S ribosomal RNA. Lower Panel: Western blot of total proteins extracted from different strains tested. TBSV p33 and p92 tagged with His6-tag were detected with an anti-HIS antibody. Total proteins were stained with Ponceau S on PVDF membrane after transfer. Each experiment was repeated.

**S1 Video**

**Recruitment of *Arabidopsis* Rab5B into the tombusvirus replication compartment in *N. benthamiana*.** Confocal laser microscopy shows partial co-localization of TBSV RFP-tagged p33 replication protein with the GFP-AtRab5B protein in *N. benthamiana* cells. Transiently expressed GFP-AtRab5B and TBSV p33-RFP are visualized by confocal microscopy. The merged images are shown in the bottom two panels.

**S2 Video**

**Recruitment of *Arabidopsis* Rab5-positive endosomes into the TBSV replication compartment through actin filaments in *N. benthamiana*.** Confocal laser microscopy shows partial co-localization of TBSV BFP-tagged p33 replication protein with the RFP-AtRab5B protein and actin filaments (detected via GFP-mTallin) in *N. benthamiana* cells. Note that the TBSV replication compartment is known to include peroxisomes.

**S3 Video**

**Recruitment of *Arabidopsis* Rab5-positive endosomes into the CIRV replication compartment through actin filaments in *N. benthamiana*.** Confocal laser microscopy shows partial co-localization of CIRV BFP-tagged p36 replication protein with the RFP-AtRab5B protein and actin filaments (detected via GFP-mTallin) in *N. benthamiana* cells. Note that the CIRV replication compartment is known to include mitochondria.
